# Identification of significant genes and therapeutic agents for breast cancer by integrated genomics

**DOI:** 10.1080/21655979.2021.1931642

**Published:** 2021-06-21

**Authors:** Xiao Sun, Zhenzhen Luo, Liuyun Gong, Xinyue Tan, Jie Chen, Xin Liang, Mengjiao Cai

**Affiliations:** Department of Oncology, The First Affiliated Hospital, Xi’an Jiaotong University, Xi’an, Shanxi P.R. China

## Abstract

Breast cancer is the most commonly diagnosed malignancy in women; thus, more cancer prevention research is urgently needed. The aim of this study was to predict potential therapeutic agents for breast cancer and determine their molecular mechanisms using integrated bioinformatics. Summary data from a large genome-wide association study of breast cancer was derived from the UK Biobank. The gene expression profile of breast cancer was from the Oncomine database. We performed a network-wide association study and gene set enrichment analysis to identify the significant genes in breast cancer. Then, we performed Gene Ontology analysis using the STRING database and conducted Kyoto Encyclopedia of Genes and Genomes pathway analysis using Cytoscape software. We verified our results using the Gene Expression Profile Interactive Analysis, PROgeneV2, and Human Protein Atlas databases. Connectivity map analysis was used to identify small-molecule compounds that are potential therapeutic agents for breast cancer. We identified 10 significant genes in breast cancer based on the gene expression profile and genome-wide association study. A total of 65 small-molecule compounds were found to be potential therapeutic agents for breast cancer.

## Graphical Abstract

**Figure:**
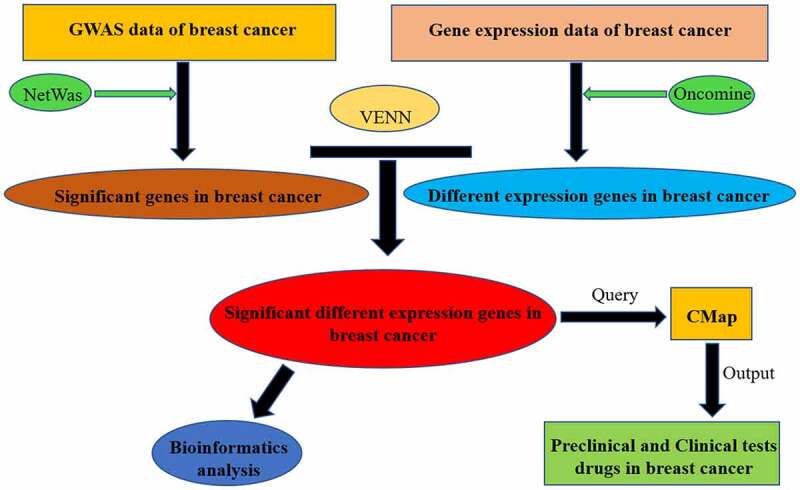

## 1. Introduction

Breast cancer is a frequently diagnosed cancer in women with a family history [1]. Breast cancer is a heterogeneous disease with different molecular subtypes and biological behaviors. Gene microarray technology and immunohistochemical techniques have classified breast cancers into different types [2]. The estrogen receptor (ER) is the most important prognostic and predictive immunohistochemical marker in breast cancer. ER-negative tumors tend to be of higher histological grade, are more sensitive to chemotherapy, and are more likely to metastasize to visceral organs [3,4]. Breast cancer does not have a poor prognosis, and there is no lack of therapeutic targets. ER positive tumors represent about 70% of all breast cancers and there are a lot of therapeutic targets, as well as for HER2 positive breast cancer (about 20% of all BC). The only subtype lacking for target therapies is the triple negative subtype [5,6]. There is an urgent need to find available drugs and clarify their molecular mechanisms in breast cancer treatment.

Most previous studies have focused on identifying novel prognostic markers and drug targets for breast cancer [7–9]. Sulaiman *et al*. [10] reported that a synthetic azaspirane targets the Janus kinase/signal transducer and activation of transcription 3 pathway in breast cancer. Huang *et al*. [11] demonstrated that the Gαh-PLCδ1 signaling axis drives metastatic progression in breast cancer. However, due to toxicity, cost, the chemical effects of novel prognostic markers and drug targets for breast cancer that need further research [12], not all previous findings contribute to breast cancer treatment; breast cancer still lacks therapeutic targets and with poorer prognosis. And there is still an urgent need to identify additional therapeutic and prognostic targets in breast cancer [13].

Genome-wide association studies (GWAS) are widely used to characterize the genetic mechanisms that underlie complex diseases. Integrative analyses of GWAS data are rapidly becoming a standard approach to explore the genetic basis of disease susceptibility [14]. Network-wide association studies (NetWAS) can identify relevant disease-gene associations by integrating tissue-specific networks and GWAS results [15,16]. Prior studies have shown that the network-associated analysis of GWAS data is highly efficient when used to identify novel causal genes of complex diseases [17,18].

In this study, to better understand the molecular mechanisms and find therapeutic agents for breast cancer, we identified novel candidate therapeutic agents for breast cancer treatment by integrating genomic data with drug database analysis. In total, 65 small-molecule compounds were identified, including trichostatin A, LY-294,002, econazole, prestwick-1082, and vorinostat. Our study demonstrates the usefulness of this approach for evaluating the relationship among genes, diseases, and drugs. These findings will pave the way for the discovery of potential therapeutic targets for breast cancer.

## 2. Methods

### 2.1 Summary of GWAS datasets in breast cancer

The UK Biobank is a large, population-based prospective UK study, which was established to identify genetic and nongenetic determinants of various diseases. It comprises approximately 500,000 individuals with extensively detailed phenotypes. Their genotypes were determined using an array that included 847,441 genetic polymorphisms, enabling the identification of novel genetic variants in a uniformly genotyped and phenotyped cohort of unprecedented size [19]. Using data from the UK Biobank, samples from the participants were genotyped on the UK Biobank Axiom array and UK BiLEVE custom array. Genotype imputation was conducted with IMPUTE software against the UK10K haplotype panel and the 1000 Genomes Project phase 3 panel. GWAS analysis was performed by SNPTEST using a logistic regression model. A genome-wide gene-association study was performed using the MAGMA gene analysis tool, and multiple genes and genetic variants were identified. The Icelandic GWAS dataset from the deCODE Genetics genealogical database was based on whole-genome sequencing using Illumina technology. Finally, meta-analysis of small nucleotide polymorphisms (SNPs) in the UK Biobank and deCODE sample was performed using the METAL analysis tool [20].

The atlas of genetic associations in the UK Biobank (GeneATLAS, http://geneatlas.roslin.ed.ac.uk) helps researchers effectively analyze UK Biobank results without high computational costs. It also allows users to query genome-wide association results for 9,113,133 genetic variants and download GWAS summary statistics for more than 30 million imputed genetic variants (>23 billion phenotype–genotype pairs) [21]. We downloaded large-scale GWAS breast cancer summary data from the atlas of genetic associations. Detailed descriptions of sample characteristics, experimental designs, statistical analyses, and quality control can be found in previous studies.

### 2.2 Gene expression datasets

Oncomine (https://www.oncomine.org) is a cancer microarray database and web-based data mining platform for facilitating discovery. In this study, differentially expressed genes (DEGs) in breast cancer were identified by comparing cancer samples to respective normal samples using the Oncomine database. The heatmap of significant DEGs in breast cancer was driven from the Oncomine.

### 2.3 Identification of significant genes in breast cancer

NetWAS (https://hb.flatironinstitute.org/netwas/) integrates tissue-specific networks and significant GWAS association results, and identifies relevant disease-gene associations based on genomics. Briefly, SNP-level association statistics were converted into gene-level statistics (gene-based *P* values), which then were integrated with tissue-specific networks to predict the causal genes [18]. Greene *et al*. [13] demonstrated that NetWAS is more accurate than GWAS alone. In this study, we identified the most relevant genes in breast cancer using NetWAS.

### 2.4 Kyoto Encyclopedia of Genes and Genomes pathway and Gene Ontology analyses

Cytoscape is one of the most successful network biology analysis and visualization tools. It exposes more than 270 core functions and 34 applications as REST-callable functions with standardized JSON interfaces supported by Swagger documentation [22]. CluePedia, a plug-in in Cytoscape, can search for certain Kyoto Encyclopedia of Genes and Genomes (KEGG) signaling pathways of certain genes by calculating linear and nonlinear statistical dependencies from experimental data [23]. KEGG signaling pathways were identified by CluePedia. Search Tool for the Retrieval of Interacting Genes (STRING) (https://string-db.org/cgi/input.pl) is an online tool that for Gene ontology (GO) analysis in gene sets [24,25]. GO is a commonly used bioinformatics tool that provides comprehensive information on the gene function of individual genomic products based on defined features consisting of three domains: biological process (BP), cellular component (CC), and molecular function (MF) [26]. We conducted GO analysis using the STRING database.

### 2.5 Analysis of the correlation between significant genes and breast cancer

Gene Expression Profiling Interactive Analysis (GEPIA, http://gepia.cancer-pku.cn) is a web server for analyzing RNA-sequencing expression data of 9,736 tumors and 8,587 normal samples from The Cancer Genome Atlas and Genotype-Tissue Expression projects, using a standard processing pipeline [27]. The Human Protein Atlas (HPA, www.proteinatlas.org) is an immunohistochemistry-based map of protein expression profiles in normal tissues, cancer tissues, and cell lines, and provides a resource for pathology-based biomedical research, including protein biomarker discovery [28–30]. Correlations between significant genes and breast cancers were analyzed with GEPIA and HPA.

### 2.6 Analysis of the correlation between significant gene expression and overall survival

PROGgeneV2 (http://www.compbio.iupui.edu/proggene), a tool that can be used to predict the prognostic implication of genes in cancers, is written in PHP5 with a MySQL database backend, which stores gene expression data, covariates data, and metadata for cataloged studies in the form of relational database tables. Survival analysis in PROGgeneV2 is done using the backend R script; users can input multiple genes and use combined analysis to create survival plots for different genes of interest [31]. We used PROGgeneV2 to analyze the relationship between overall survival and genes that were overexpressed and underexpressed in breast cancer.

### 2.7 Drug prediction analysis

CMap (https://portals.broadinstitute.org/cmap/) is a collection of genome-wide transcriptional expression data from cultured human cells treated with bioactive small molecules and simple pattern-matching algorithms that together enable the discovery of functional connections among drugs, genes, and diseases through the transitory feature of common gene expression changes [32–34]. We used CMap to identify small-molecule compounds as potential therapeutic agents to target the significant genes in breast cancer.

## 3. Results

### 3.1 Identification of significant DEGs in breast cancer

**Figure 1.**
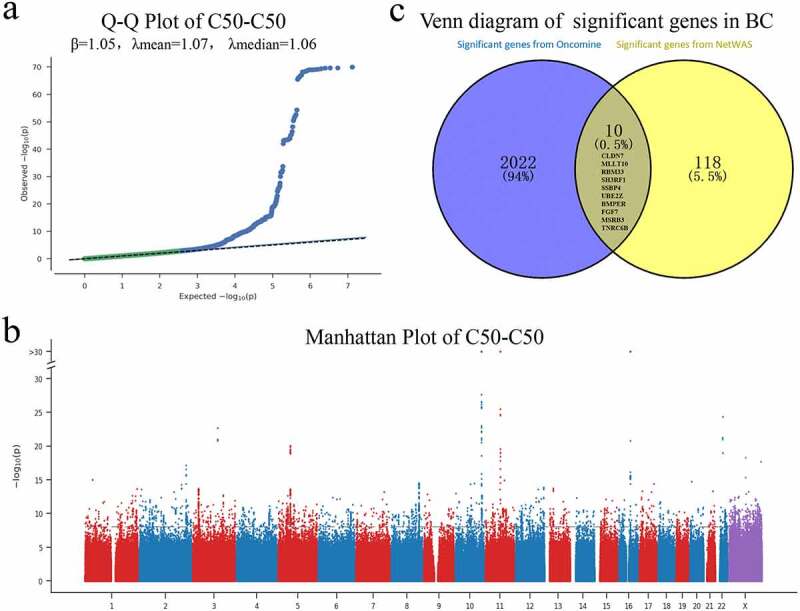
A) Q-Q Plot of C50-C50 (β = 1.05, λmean = 1.07, λmedian = 1.06) Containing 9,113,133 imputed variants that passed quality control (QC), with a P different than 0; b) Manhattan PLOT (IMPUTED) CONTAINING all (QC and non-QC) 30,798,054 imputed variants; c) Venn diagram of significant genes (CLDN7,MLLT10,RBM33,SH3RF1,SSBP4, UBE2Z,BMPER,FGF7,MSRB3,TNRC6B) in breast cancer (BC)

To identify the significant DEGs in breast cancer, we retrieved GWAS summary data (C50-C50) of breast cancer from the UK Biobank, and microarray expression profiles of breast cancer from the Oncomine database. C50-C50 contained 10,478 malignant neoplasm of breast cases and 235,016 controls for the analyses, and the data were consolidated and normalized (Figure 1(a,b)).

**Figure 2.**
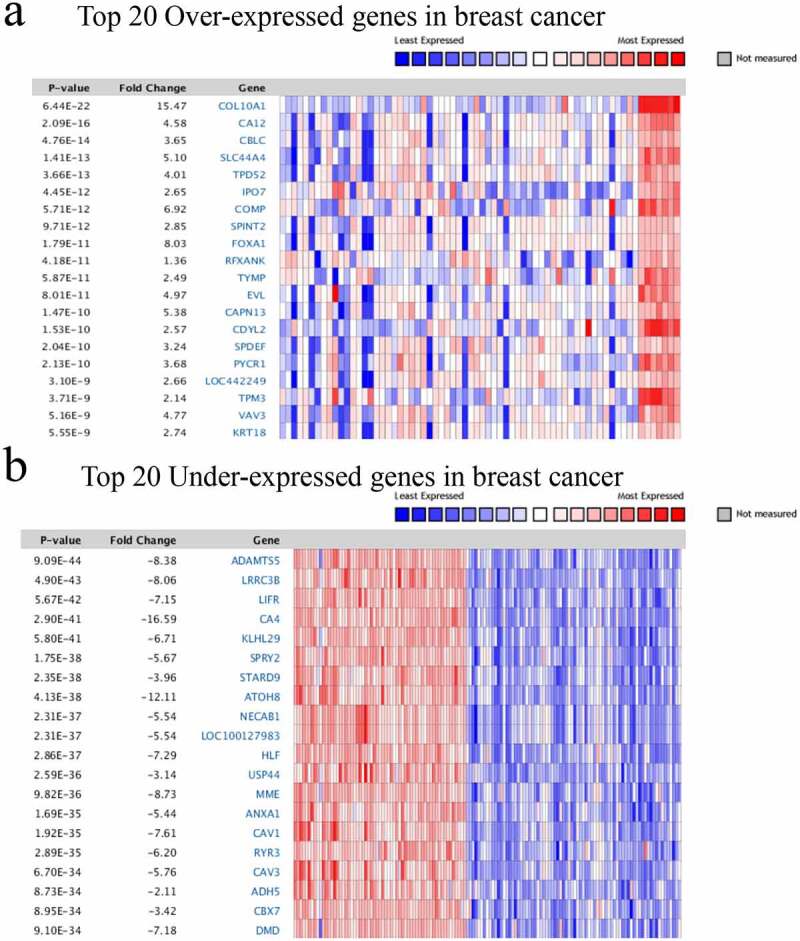
Heatmap of significant different expression genes in BC. a)Top 20 Over-expressed genes; b) Top 20 Under-expressed genes

From NetWAS of GWAS summary data, we converted SNP-level association statistics into gene-level statistics (gene-based *P* values) and identified the 127 most relevant genes in breast cancer (Table 1). A total of 1019 overexpressed genes (Supplementary Figure 1) and 1019 underexpressed genes (Supplementary Figure 2) were identified by Oncomine. The top 20 DEGs in breast cancer compared to the normal controls are shown in a heatmap (Figure 2).

**Table 1.** Significant genes in breast cancer identified using NetWAS

Gene symbol	Training label	NetWAS Score
DVL2	1	0.235936
SNF8	1	0.174439
ADSL	1	0.16157
OR2AE1	1	0.160822
ACADVL	1	0.160651
OR2A5	1	0.149804
TRIM4	1	0.148613
GJC3	1	0.146056
TMEM161A	1	0.143088
ITGA5	1	0.128071
SPDYE3	1	0.120586
LRRD1	1	0.116357
PHF23	1	0.111639
ATG9B	1	0.106706
DIDO1	1	0.105009
KRIT1	1	0.103645
CCND1	1	0.100449
SAP30BP	1	0.100171
GRID2IP	1	0.099812
MAFK	1	0.096834
SLC25A17	1	0.094211
TMEM184A	1	0.093676
TSC22D4	1	0.093096
SCAMP2	1	0.093005
ZCWPW1	1	0.092518
LRP1	1	0.088344
PEG10	1	0.085924
RBM33	1	0.084988
FNIP2	1	0.082357
TNRC6B	1	0.080609
MUC17	1	0.080398
MUC12	1	0.070727
C7orf61	1	0.069049
CSK	1	0.065686
AVIL	1	0.064848
FGFR2	1	0.062894
OR12D2	1	0.062179
PILRB	1	0.061788
PDLIM4	1	0.060831
AZGP1	1	0.060558
WNT2	1	0.058427
MMD	1	0.057349
CARS	1	0.05583
ATP6V0A4	1	0.055112
BMPER	1	0.054279
MPV17L2	1	0.053732
RBM48	1	0.053696
P4HA2	1	0.050581
PHLDA2	1	0.050163
NAP1L4	1	0.049917
NR2F6	1	0.048975
UBE2Z	1	0.048287
ISYNA1	1	0.048024
CYP51A1	1	0.047283
CYP2S1	1	0.047158
MEPCE	1	0.046038
GIP	1	0.045434
CCS	1	0.043749
GDF15	1	0.041477
KCNN4	1	0.041465
NYAP1	1	0.041088
SLC2A4	1	0.040597
RBM39	1	0.040581
SH3RF1	1	0.039898
NPLOC4	1	0.039647
PIDD1	1	0.038797
MIEF1	1	0.038407
TFAP2D	1	0.037515
PARD6B	1	0.034994
POTEJ	1	0.0348
FNDC1	1	0.034535
DLX2	1	0.033885
NEUROG3	1	0.033203
SPEG	1	0.031537
RASSF3	1	0.03124
CBX7	1	0.029876
ELOF1	1	0.028148
WNT3	1	0.027907
IGFBP2	1	0.026449
SPTBN2	1	0.02607
DLL4	1	0.024507
FZD7	1	0.024165
PLCE1	1	0.023692
KLRC4	1	0.023357
PSTK	1	0.023215
ASIC4	1	0.022743
NEUROD4	1	0.021315
ZDHHC24	1	0.018987
RSPO3	1	0.018473
SLC35D3	1	0.016828
ADCY3	1	0.016417
ABCG2	1	0.016354
DNAH9	1	0.016198
SMIM5	1	0.015532
CTDNEP1	1	0.015522
C12orf80	1	0.014825
FAM71E2	1	0.014777
GPRIN2	1	0.014572
SSBP4	1	0.014453
DLG4	1	0.01316
USHBP1	1	0.013133
CLDN7	1	0.012219
ZNF18	1	0.010693
TTLL6	1	0.00984
KCNK17	1	0.009442
PHF20	1	0.009367
TNP1	1	0.009195
ZBTB2	1	0.008477
RBM43	1	0.007998
PNLIP	1	0.007244
CASC10	1	0.007176
ELL	1	0.006518
SKIDA1	1	0.00598
MLLT10	1	0.00583
ODF3L1	1	0.005735
SPAG4	1	0.00503
TAAR8	1	0.003711
UBALD2	1	0.003033
LMAN1L	1	0.002579
LYPD5	1	0.002363
FGF7	1	0.001942
MSRB3	1	0.001837
SLC22A5	1	0.001425
ZNF420	1	0.000842
BAZ2A	1	0.000831
RNF175	1	0.000786
BBS1	1	0.000378

After overlapping the 127 most relevant genes with the 2038 DEGs in breast cancer, we identified 10 significant genes (*CLDN7, MLLT10, RBM33, SH3RF1, SSBP4, UBE2Z, BMPER, FGF7, MSRB3*, and *TNRC6B*) in breast cancer (Figure 1c). Among them, *CLDN7, MLLT10, RBM33, SH3RF1, SSBP4*, and *UBE2Z* were overexpressed; and *BMPER, FGF7, MSRB3*, and *TNRC6B* were underexpressed.

### 3.2 GO and KEGG enrichment analyses of significant DEGs in breast cancer

**Figure 3:**
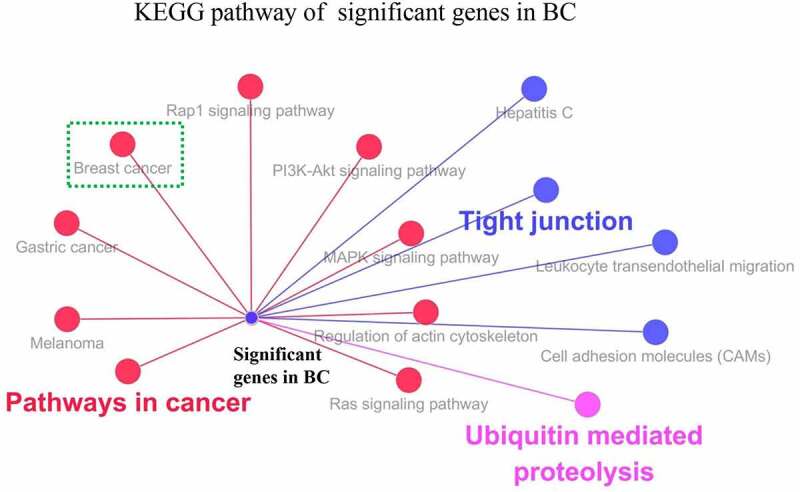
KEGG pathway analysis of significant genes (CLDN7, MLLT10, RBM33, SH3RF1, SSBP4, UBE2Z, BMPER, FGF7, MSRB3 and TNRC6B) in BC

To explore the roles of the significant DEGs in breast cancer, we played GO and KEGG enrichment analyses. BP analysis revealed that the significant genes in breast cancer were mainly enriched in the Wnt signaling pathway, calcium-modulating pathway, protein repair, gene silencing by microRNA (miRNA), mRNA cleavage involved in gene silencing by miRNA, and positive regulation of epithelial cell proliferation involved in lung morphogenesis (Table 2). MF analysis showed that significant genes were enriched in functions related to oxidoreductase activity, acting on a sulfur group of donors and disulfide as acceptor, and phosphoinositide 3-kinase (PI3K) and PIK3CA activities (Table 2). CC analysis showed that significant genes were enriched in P-bodies. KEGG analysis revealed that significant genes in breast cancer were mainly involved in pathways in cancer, breast cancer, gastric cancer, melanoma, the PI3K/Akt signaling pathway, mitogen-activated protein kinase (MAPK) signaling pathway, Ras signaling pathway, tight junctions, and ubiquitin-mediated proteolysis (Figure 3).

**Table 2.** Gene ontology (GO) enrichment result of significant genes in breast cancer

	Biological processes	
term ID	term description	false discovery rate
GO:0007223	Wnt signaling pathway, calcium modulating pathway	0.0024
GO:0030091	protein repair	0.0024
GO:0035195	gene silencing by miRNA	0.0024
GO:0035279	mRNA cleavage involved in gene silencing by miRNA	0.0024
GO:0060501	positive regulation of epithelial cell proliferation involved in lung morphogenesis	0.0024
GO:0035278	miRNA mediated inhibition of translation	0.003
GO:0060213	positive regulation of nuclear-transcribed mRNA poly(A) tail shortening	0.003
GO:0010463	mesenchymal cell proliferation	0.0039
GO:0060445	branching involved in salivary gland morphogenesis	0.0039
GO:0031069	hair follicle morphogenesis	0.0078
GO:2,000,026	regulation of multicellular organismal development	0.0082
GO:0051173	positive regulation of nitrogen compound metabolic process	0.0168
GO:0036092	phosphatidylinositol-3-phosphate biosynthetic process	0.019
GO:0010604	positive regulation of macromolecule metabolic process	0.0194
GO:0031325	positive regulation of cellular metabolic process	0.0194
GO:0048522	positive regulation of cellular process	0.0194
GO:0051254	positive regulation of RNA metabolic process	0.0194
GO:0060688	regulation of morphogenesis of a branching structure	0.0194
GO:0007267	cell-cell signaling	0.0199
GO:1,903,313	positive regulation of mRNA metabolic process	0.0242
Molecular functions		
GO:0016671	oxidoreductase activity, acting on a sulfur group of donors, disulfide as acceptor	0.0083
GO:0016303	1-phosphatidylinositol-3-kinase activity	0.0406
GO:0046934	phosphatidylinositol-4,5-bisphosphate 3-kinase activity	0.0406
Cellular components		
GO:0000932	P-body	0.0029

### 3.3 Correlation between significant DEGs and breast cancer

**Figure 4.**
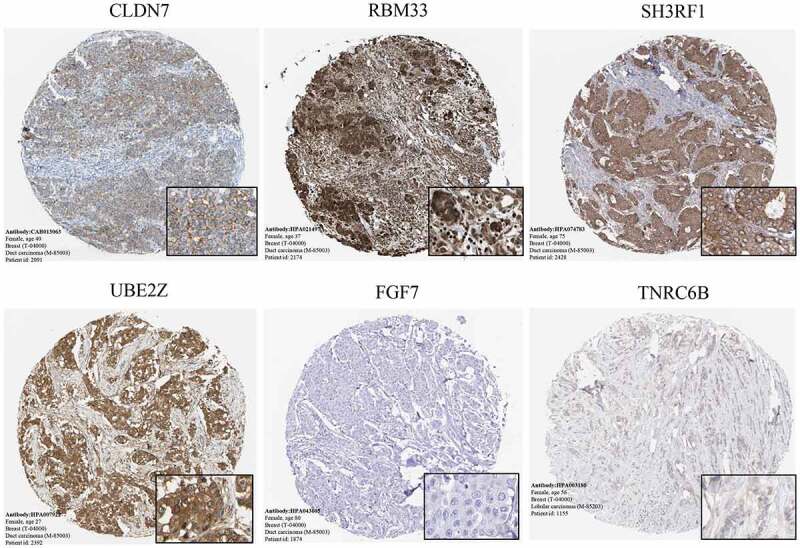
The immunohistochemistry of significant genes(CLDN7, RBM33, SH3RF1, UBE2Z, FGF7 and TNRC6B) in BC

To verify the significant DEGs of breast cancer, we further explore the DEGs. Consistent with the identification of significant genes, protein profiling in breast cancer samples from the HPA using immunohistochemistry showed that the gene expression of *CLDN7, RBM33, SH3RF1*, and *UBE2Z* was significantly enriched in breast cancer, whereas there was no significant enrichment of *FGF7* and *TNRC6B* (Figure 4).

**Figure 5.**
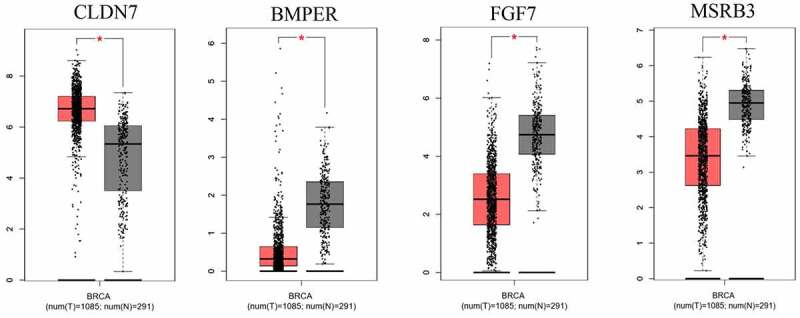
The different expression of significant genes genes(CLDN7, BMPER, FGF7 and MSRB3) in breast cancer samples to normal samples, the red box mean in breast cancer samples and the black box mean in normal samples. *:p < 0.05

The significant DEGs (*CLDN7, BMPER, FGF7, MSRB3*) in breast cancer samples compared to normal samples also showed coincident results of significant gene identification (Figure 5).

### 3.4 Correlation between overall survival and significant DEGs in breast cancer

**Figure 6.**
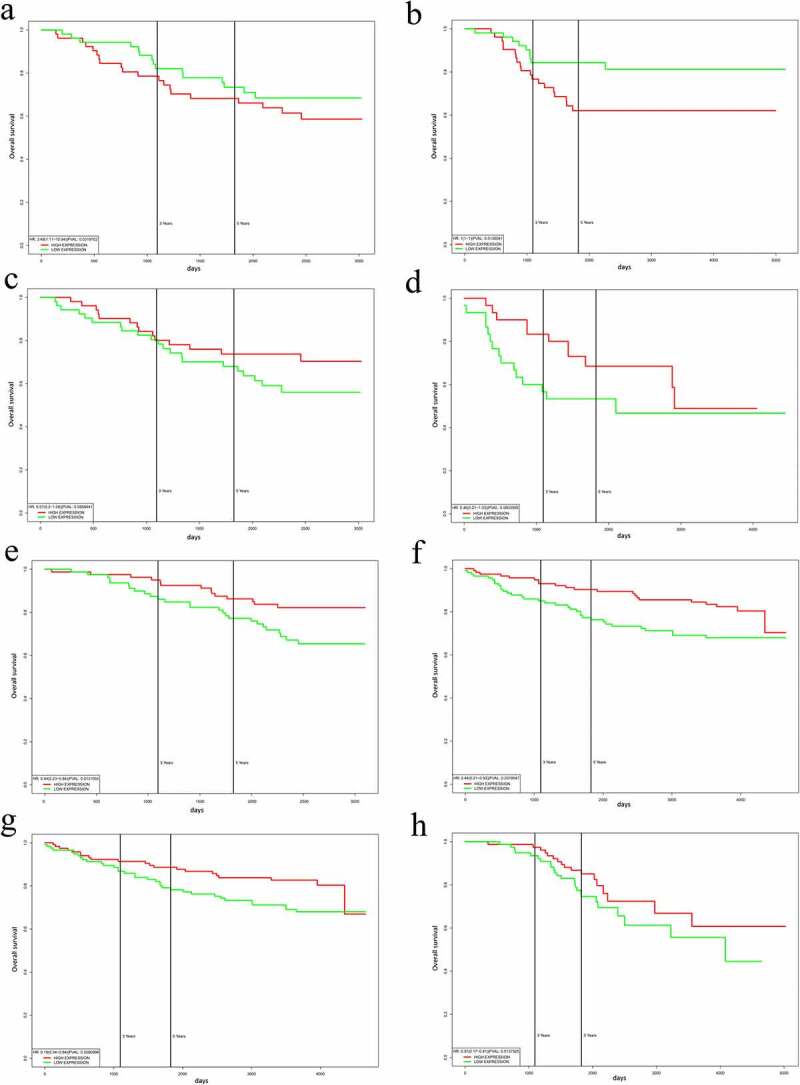
Overall survival analysis combined multiple genes expression. a)Combined significant Over-expressed genes(CLDN7, MLLT10, RBM33, SH3RF1, SSBP4 and UBE2Z) in BC based on GSE58812; b)Combined significant Over-expressed genes(CLDN7, MLLT10, RBM33, SH3RF1, SSBP4 and UBE2Z) in BC based on GSE42568; c)Combined significant under-expressed genes(BMPER, FGF7, MSRB3 and TNRC6B) in BC based on GSE42568; d)Combined BMPER,FGF7,MSRB3 and TNRC6B in BC based on GSE37751; e)Combined BMPER,MSRB3 and TNRC6B in BC based on GSE1456_U133B; f)Combined BMPER,MSRB3 and TNRC6B in BC based on GSE3494_U133B; g)Combined FGF7 and TNRC6B in BC based on GSE3494_U133A; h)Combined FGF7 in BC based on GSE9893

In the analysis of the correlation between overall survival and significant DEG expression (*CLDN7, MLLT10, RBM33, SH3RF1, SSBP4*, and *UBE2Z*) in breast cancer, we found a shorter survival time based on GSE5881 (Figure 6a) and GSE42568 (Figure 6b) (*P*< 0.05). The significantly underexpressed genes (*BMPER, FGF7, MSRB3*, and *TNRC6B*) in breast cancer were correlated with a longer survival time based on GSE42568 (Figure 6c) and GSE37751 (Figure 6d) (*P*> 0.05), this correlation cannot be demonstrated. The overall survival analysis combined significantly underexpressed genes (*BMPER, MSRB3*, and *TNRC6B*) in breast cancer based on GSE1456_U133B (Figure 6e) and GSE3494_U133B (Figure 6f); combined *FGF7* and *TNRC6B* based on GSE3494_U133A (Figure 6g); and combined single *FGF7* based on GSE9893 (Figure 6h) were also correlated with longer survival in breast cancer (*P*< 0.05).

### 3.5 Drug prediction analysis

To identify potential small-molecule compounds with therapeutic effects on breast cancer, drug prediction analysis was performed using CMap. A total of 65 drugs were predicted, and the 10 most significant were trichostatin A, LY-294,002, econazole, Prestwick-1082, vorinostat, lomefloxacin, clorsulon, amantadine, thiostrepton, and orciprenaline (Table 3).

**Table 3.** The most significant drugs provided by cmap to reverse core genes of breast cancers

cmap name	mean	enrichment	p	specificity
trichostatin A	−0.447	−0.422	0	0.3069
LY-294,002	−0.389	−0.402	0	0.1534
econazole	0.716	0.91	0.00006	0.0154
Prestwick-1082	0.749	0.949	0.00012	0
vorinostat	−0.508	−0.568	0.00038	0.2832
lomefloxacin	−0.607	−0.735	0.0007	0
clorsulon	0.714	0.85	0.00072	0
amantadine	0.659	0.838	0.00107	0.0063
thiostrepton	−0.738	−0.831	0.00147	0.037
orciprenaline	0.589	0.801	0.00298	0.0058
thiamphenicol	0.428	0.725	0.00352	0.1173
khellin	0.444	0.713	0.00459	0.0181
thiethylperazine	0.576	0.767	0.00567	0.011
felbinac	0.476	0.763	0.00599	0.0468
Chicago Sky Blue 6B	0.472	0.762	0.00605	0.0061
vinburnine	0.41	0.756	0.0068	0.0351
scriptaid	−0.688	−0.849	0.00683	0.0833
Prestwick-1103	0.338	0.747	0.00786	0.0435
naringenin	0.444	0.745	0.00796	0.0585
adiphenine	0.385	0.683	0.00811	0.2819
terazosin	0.561	0.74	0.00875	0.0112
oxolamine	0.339	0.739	0.00897	0.0385
monobenzone	−0.557	−0.736	0.00961	0.0203
chenodeoxycholic acid	0.326	0.731	0.01034	0.0495
rifabutin	−0.623	−0.823	0.01104	0.125
levonorgestrel	−0.38	−0.61	0.01158	0.0874
cinnarizine	0.413	0.72	0.01233	0.0146
oxybuprocaine	−0.563	−0.715	0.01339	0.0225
metformin	0.335	0.475	0.01352	0.0311
memantine	0.374	0.7	0.01677	0
acetohexamide	0.292	0.698	0.01719	0.0059
proxyphylline	−0.515	−0.694	0.01846	0.0792
R-atenolol	0.273	0.688	0.01969	0
quinostatin	−0.756	−0.9	0.02	0.1832
vinblastine	0.41	0.781	0.02129	0.0774
colecalciferol	−0.407	−0.684	0.02158	0.0217
dexibuprofen	−0.501	−0.681	0.02248	0.0173
BCB000040	0.616	0.679	0.02308	0.0057
levopropoxyphene	0.28	0.676	0.02401	0.0063
sulconazole	−0.396	−0.676	0.02455	0.0855
nadolol	0.518	0.673	0.02514	0.2857
hycanthone	0.514	0.67	0.02612	0.1814
CP-863,187	0.618	0.668	0.02707	0.0704
karakoline	0.317	0.557	0.02854	0.0267
etamivan	0.523	0.663	0.0292	0.0268
methylergometrine	−0.407	−0.662	0.02952	0.0357
homochlorcyclizine	0.321	0.66	0.03069	0.1087
fluorometholone	−0.401	−0.656	0.03223	0.057
probucol	−0.382	−0.549	0.03266	0.0061
phthalylsulfathiazole	−0.406	−0.591	0.03308	0.2864
flecainide	0.35	0.547	0.03329	0.0058
tetraethylenepentamine	−0.384	−0.542	0.03655	0.0229
josamycin	0.469	0.588	0.03679	0.1235
torasemide	−0.418	−0.645	0.03796	0.0545
nadide	0.511	0.637	0.04225	0.0643
fosfosal	0.459	0.637	0.04265	0.0142
rescinnamine	−0.592	−0.723	0.04363	0.0248
diethylcarbamazine	0.396	0.634	0.04416	0.0252
cyclopentolate	−0.499	−0.632	0.0444	0.05
harmol	0.275	0.634	0.04444	0.0853
metixene	0.288	0.632	0.04534	0.1593
idoxuridine	0.474	0.572	0.0458	0.0838
LM-1685	−0.571	−0.714	0.04795	0.0238
iohexol	0.253	0.628	0.04808	0.1657
fenbendazole	0.497	0.626	0.0489	0.11

## 4. Discussion

Breast cancer is the most commonly diagnosed malignancy in women worldwide and is the main cause of cancer-related death in women [35–37]. Although there are a lot of effective therapeutic agents for breast cancer, breast cancer remains a major health problem and is a top biomedical research priority [38–40], as there is an urgent need for effective breast cancer treatments.

In this study, we identified 10 significant genes (CLDN7, MLLT10, RBM33, SH3RF1, SSBP4, UBE2Z, BMPER, FGF7, MSRB3, and TNRC6B) in breast cancer using combined GWAS data and profiling of DEGs. Protein profiling in breast cancer samples from the HPA using immunohistochemistry and analysis of significant DEGs in breast cancer samples compared to normal samples from GEPIA further verified the results. Significantly overexpressed genes (*CLDN7, MLLT10, RBM33, SH3RF1, SSBP4*, and *UBE2Z*) were correlated with shorter survival, whereas underexpressed genes (*BMPER, FGF7, MSRB3*, and *TNRC6B*) were correlated with longer survival in breast cancer.

Consistent with our findings, previous studies have shown that some of these genes play important roles in the development of breast cancer. For example, Bernardi *et al*. [41] showed that *CLDN7* is associated with a shorter time to recurrence, suggesting its contribution to the aggressiveness of breast cancer. In a GWAS, Guo *et al*. [42] identified common genetic loci for breast cancer risk including SSBP4. Whole transcriptome analysis by Bauer *et al*. [43] demonstrated that BMPER plays a possible therapeutic role in breast cancer. Fu *et al*. [44] demonstrated that acetylation, expression and recruitment of FGF7 promoters induce cancer growth and progression. Zhu *et al*. [45] found that targeting *FGF7* can exert oncogenic functions in breast cancer. A previous study showed that the ZEB1-MSRB3 axis is related to breast cancer genome stability [46]. Interestingly, other DEGs in breast cancer identified in this study, including *MLLT10, RBM33, SH3RF1, UBE2Z*, and *TNRC6B*, have not been proven in previous studies. We believe that these are potentially novel key genes in breast cancer.

BP analysis in GO annotation indicated that the 10 significant genes are mainly enriched in the Wnt signaling pathway, which plays an important role in the occurrence and development of many cancers. Inhibiting this pathway can suppress breast cancer growth and metastasis [47–49]. MF analysis of GO suggested that the DEGs were most significantly enriched in functions related to oxidoreductase activity. The redox reaction is accompanied by tumor development. CC analysis of GO annotation showed that the 10 DEGs were enriched in P-bodies. A previous study suggested that P-body disassembly correlates with breast cancer progression [50].

KEGG analysis of the 10 DEGs showed their enrichment in breast cancer, gastric cancer, melanoma, the PI3K/Akt signaling pathway, MAPK signaling pathway, Ras signaling pathway, tight junctions, and ubiquitin-mediated proteolysis. Some of these pathways contribute to the development of breast cancer. For example, the PI3K pathway is found in many types of cancer and plays an important role in breast cancer cell proliferation [51]. Ras signaling is a key determinant of poor survival in breast cancer patients [52]. Abnormal MAPK signaling plays a core role in the regulation of growth and survival, and the development of drug resistance in triple-negative breast cancer [53].

The aim of this work was to identify significant genes and potential therapeutic agents for breast cancer based on genomics. We found 65 potentially small-molecule compounds to reverse significant genes in breast cancer. The 10 most significant drugs were trichostatin A, LY-294,002, econazole, Prestwick-1082, vorinostat, lomefloxacin, clorsulon, amantadine, thiostrepton, and orciprenaline. Consistent, with our study, it has been reported that trichostatin A, a histone deacetylase inhibitor, has therapeutic potential in breast cancer [54]. Jiang *et al*. [55] showed that trichostatin A sensitizes ER-negative breast cancer cells to tamoxifen. LY294002, a specific inhibitor of the PI3K pathway, can decrease the rate of cell growth and increase the therapeutic sensitivity in MCF7 cells expressing wild-type p53, which may be useful for the treatment of breast cancer [56]. Econazole is a novel PI3K/AKT signaling pathway inhibitor, which can be used to overcome adriamycin resistance and improve chemotherapy sensitivity in breast cancer [57]. A preclinical study showed that vorinostat can prevent the formation of brain metastases in breast cancer [58]. Yang *et al*. [59] suggested that thiostrepton is a promising agent for triple-negative breast cancer. Kwok *et al*. [60] showed that thiostrepton selectively targets breast cancer cells through inhibition of Forkhead box M1 expression. However, some of the predicted drugs, such as Prestwick-1082, lomefloxacin, clorsulon, amantadine, and orciprenaline, have not been shown to directly play a role in breast cancer. Thus, future studies are needed to confirm our findings.

Compared to previous studies [61–63], we conducted an analysis combining genomic data with drug database analysis to identify novel candidate therapeutic agents for breast cancer treatment. Our study demonstrates the usefulness of this approach for evaluating the relationship among genes, diseases, and drugs. These findings will pave the way for the discovery of potential therapeutic targets for breast cancer.

## 5. Conclusion:

Combined analyses of network-wide association studies, gene expression profiles, and drug databases are helpful for identifying potential therapeutic agents for diseases. This method is a new paradigm that can guide future research directions.

## Supplementary Material

Supplemental Material

## Data Availability

All materials are available by the corresponding author.

## Highlights

1. Combined analyses of network-wide association studies, gene expression profiles, and drug databases.
2. A useful approach for evaluating the relationship among genes, diseases, and drugs.

These findings will pave the way for the discovery of potential therapeutic targets for breast cancer.
