# Supplementary material for: Identification of significant genes and therapeutic agents for breast cancer by integrated genomics
Source: Bioengineered. 2021 Jun 21;12(1):2140–54. doi: 10.1080/21655979.2021.1931642 (PMC8806825; doi:10.1080/21655979.2021.1931642)
Supplement: Supplemental Material [file KBIE_A_1931642_SM7001.zip › supplementary1.docx]

**Top 5% Over-expressed genes in breast cancer**

| Gene symbol | Gene name |
| --- | --- |
| AAGAB | alpha- and gamma-adaptin binding protein |
| ABCB8 | ATP-binding cassette, sub-family B (MDR/TAP), member 8 |
| ABCC5 | ATP-binding cassette, sub-family C (CFTR/MRP), member 5 |
| ABHD2 | abhydrolase domain containing 2 |
| ABL1 | c-abl oncogene 1, receptor tyrosine kinase |
| ACTRT2 | actin-related protein T2 |
| ADAL | adenosine deaminase-like |
| ADAM19 | ADAM metallopeptidase domain 19 (meltrin beta) |
| ADAM8 | ADAM metallopeptidase domain 8 |
| ADAMTSL2 | ADAMTS-like 2 |
| ADAR | adenosine deaminase, RNA-specific |
| AEN | apoptosis enhancing nuclease |
| AGAP2 | ArfGAP with GTPase domain, ankyrin repeat and PH domain 2 |
| AGMAT | agmatine ureohydrolase (agmatinase) |
| AGPAT3 | 1-acylglycerol-3-phosphate O-acyltransferase 3 |
| AGR3 | anterior gradient homolog 3 (Xenopus laevis) |
| AHSG | alpha-2-HS-glycoprotein |
| AK2 | adenylate kinase 2 |
| AKR1A1 | aldo-keto reductase family 1, member A1 (aldehyde reductase) |
| ALDH18A1 | aldehyde dehydrogenase 18 family, member A1 |
| ALG3 | asparagine-linked glycosylation 3, alpha-1,3- mannosyltransferase homolog (S. cerevisiae) |
| ALKBH1 | alkB, alkylation repair homolog 1 (E. coli) |
| ALKBH5 | alkB, alkylation repair homolog 5 (E. coli) |
| AMFR | autocrine motility factor receptor |
| AMMECR1L | AMME chromosomal region gene 1-like |
| ANKRD28 | ankyrin repeat domain 28 |
| ANKRD39 | ankyrin repeat domain 39 |
| ANKRD5 | ankyrin repeat domain 5 |
| ANLN | anillin, actin binding protein |
| ANXA7 | annexin A7 |
| AP2B1 | adaptor-related protein complex 2, beta 1 subunit |
| AP2S1 | adaptor-related protein complex 2, sigma 1 subunit |
| AP3M2 | adaptor-related protein complex 3, mu 2 subunit |
| APOL2 | apolipoprotein L, 2 |
| APOO | apolipoprotein O |
| APOOL | apolipoprotein O-like |
| AQP2 | aquaporin 2 (collecting duct) |
| AQP8 | aquaporin 8 |
| ARHGDIA | Rho GDP dissociation inhibitor (GDI) alpha |
| ARHGEF16 | Rho guanine nucleotide exchange factor (GEF) 16 |
| ARHGEF19 | Rho guanine nucleotide exchange factor (GEF) 19 |
| ARHGEF9 | Cdc42 guanine nucleotide exchange factor (GEF) 9 |
| ARID1A | AT rich interactive domain 1A (SWI-like) |
| ARL14 | ADP-ribosylation factor-like 14 |
| ARL6 | ADP-ribosylation factor-like 6 |
| ARL8A | ADP-ribosylation factor-like 8A |
| ARMC1 | armadillo repeat containing 1 |
| ARPC1B | actin related protein 2/3 complex, subunit 1B, 41kDa |
| ARTN | artemin |
| ARX | aristaless related homeobox |
| ASB7 | ankyrin repeat and SOCS box-containing 7 |
| ASH1L | ash1 (absent, small, or homeotic)-like (Drosophila) |
| ASH2L | ash2 (absent, small, or homeotic)-like (Drosophila) |
| ASNS | asparagine synthetase (glutamine-hydrolyzing) |
| ASPHD2 | aspartate beta-hydroxylase domain containing 2 |
| ASPM | asp (abnormal spindle) homolog, microcephaly associated (Drosophila) |
| ASXL2 | additional sex combs like 2 (Drosophila) |
| ATAD3B | ATPase family, AAA domain containing 3B |
| ATAD5 | ATPase family, AAA domain containing 5 |
| ATCAY | ataxia, cerebellar, Cayman type |
| ATG12 | ATG12 autophagy related 12 homolog (S. cerevisiae) |
| ATG4B | ATG4 autophagy related 4 homolog B (S. cerevisiae) |
| ATP11A | ATPase, class VI, type 11A |
| ATP1B2 | ATPase, Na+/K+ transporting, beta 2 polypeptide |
| ATP5G3 | ATP synthase, H+ transporting, mitochondrial F0 complex, subunit C3 (subunit 9) |
| ATP5J2 | ATP synthase, H+ transporting, mitochondrial F0 complex, subunit F2 |
| ATP5SL | ATP5S-like |
| ATP6V1E2 | ATPase, H+ transporting, lysosomal 31kDa, V1 subunit E2 |
| ATP8B1 | ATPase, aminophospholipid transporter, class I, type 8B, member 1 |
| ATPAF2 | ATP synthase mitochondrial F1 complex assembly factor 2 |
| AURKA | aurora kinase A |
| AURKB | aurora kinase B |
| AVL9 | AVL9 homolog (S. cerevisiase) |
| AXIN1 | axin 1 |
| B3GNT7 | UDP-GlcNAc:betaGal beta-1,3-N-acetylglucosaminyltransferase 7 |
| B3GNT8 | UDP-GlcNAc:betaGal beta-1,3-N-acetylglucosaminyltransferase 8 |
| BAIAP2L1 | BAI1-associated protein 2-like 1 |
| BAK1 | BCL2-antagonist/killer 1 |
| BCAP29 | B-cell receptor-associated protein 29 |
| BCAS4 | breast carcinoma amplified sequence 4 |
| BCAT1 | branched chain amino-acid transaminase 1, cytosolic |
| BIRC5 | baculoviral IAP repeat-containing 5 |
| BMP10 | bone morphogenetic protein 10 |
| BPIL2 | bactericidal/permeability-increasing protein-like 2 |
| BRAF | v-raf murine sarcoma viral oncogene homolog B1 |
| BRCA1 | breast cancer 1, early onset |
| BRF2 | BRF2, subunit of RNA polymerase III transcription initiation factor, BRF1-like |
| BRSK1 | BR serine/threonine kinase 1 |
| BUB1 | budding uninhibited by benzimidazoles 1 homolog (yeast) |
| BZW1L1 | basic leucine zipper and W2 domains 1 like 1 |
| C11orf82 | chromosome 11 open reading frame 82 |
| C12orf42 | chromosome 12 open reading frame 42 |
| C12orf66 | chromosome 12 open reading frame 66 |
| C14orf34 | chromosome 14 open reading frame 34 |
| C14orf68 | chromosome 14 open reading frame 68 |
| C16orf59 | chromosome 16 open reading frame 59 |
| C17orf107 | chromosome 17 open reading frame 107 |
| C17orf28 | chromosome 17 open reading frame 28 |
| C17orf39 | chromosome 17 open reading frame 39 |
| C17orf67 | chromosome 17 open reading frame 67 |
| C17orf89 | chromosome 17 open reading frame 89 |
| C17orf90 | chromosome 17 open reading frame 90 |
| C17orf99 | chromosome 17 open reading frame 99 |
| C18orf45 | chromosome 18 open reading frame 45 |
| C19orf21 | chromosome 19 open reading frame 21 |
| C19orf28 | chromosome 19 open reading frame 28 |
| C1orf113 | chromosome 1 open reading frame 113 |
| C1orf135 | chromosome 1 open reading frame 135 |
| C1orf161 | chromosome 1 open reading frame 161 |
| C1orf167 | chromosome 1 open reading frame 167 |
| C1orf172 | chromosome 1 open reading frame 172 |
| C1orf91 | chromosome 1 open reading frame 91 |
| C20orf112 | chromosome 20 open reading frame 112 |
| C20orf144 | chromosome 20 open reading frame 144 |
| C20orf20 | chromosome 20 open reading frame 20 |
| C20orf54 | chromosome 20 open reading frame 54 |
| C22orf9 | chromosome 22 open reading frame 9 |
| C2CD4A | C2 calcium-dependent domain containing 4A |
| C2orf29 | chromosome 2 open reading frame 29 |
| C3orf21 | chromosome 3 open reading frame 21 |
| C3orf49 | chromosome 3 open reading frame 49 |
| C4A | complement component 4A (Rodgers blood group) |
| C4B | complement component 4B (Chido blood group) |
| C5orf22 | chromosome 5 open reading frame 22 |
| C5orf34 | chromosome 5 open reading frame 34 |
| C6orf136 | chromosome 6 open reading frame 136 |
| C6orf64 | chromosome 6 open reading frame 64 |
| C6orf97 | chromosome 6 open reading frame 97 |
| C9orf44 | chromosome 9 open reading frame 44 |
| C9orf98 | chromosome 9 open reading frame 98 |
| CA12 | carbonic anhydrase XII |
| CABYR | calcium binding tyrosine-(Y)-phosphorylation regulated |
| CACNG4 | calcium channel, voltage-dependent, gamma subunit 4 |
| CALM3 | calmodulin 3 (phosphorylase kinase, delta) |
| CAPN13 | calpain 13 |
| CAPRIN1 | cell cycle associated protein 1 |
| CARD14 | caspase recruitment domain family, member 14 |
| CASC4 | cancer susceptibility candidate 4 |
| CASC5 | cancer susceptibility candidate 5 |
| CASK | calcium/calmodulin-dependent serine protein kinase (MAGUK family) |
| CASKIN1 | CASK interacting protein 1 |
| CASP2 | caspase 2, apoptosis-related cysteine peptidase |
| CBLC | Cas-Br-M (murine) ecotropic retroviral transforming sequence c |
| CBS | cystathionine-beta-synthase |
| CC2D1B | coiled-coil and C2 domain containing 1B |
| CCDC24 | coiled-coil domain containing 24 |
| CCDC30 | coiled-coil domain containing 30 |
| CCDC88C | coiled-coil domain containing 88C |
| CCDC93 | coiled-coil domain containing 93 |
| CCL25 | chemokine (C-C motif) ligand 25 |
| CCNB2 | cyclin B2 |
| CCNF | cyclin F |
| CCT6A | chaperonin containing TCP1, subunit 6A (zeta 1) |
| CCT6P3 | chaperonin containing TCP1, subunit 6 (zeta) pseudogene 3 |
| CCT8 | chaperonin containing TCP1, subunit 8 (theta) |
| CD22 | CD22 molecule |
| CD276 | CD276 molecule |
| CD80 | CD80 molecule |
| CDC20 | cell division cycle 20 homolog (S. cerevisiae) |
| CDC25C | cell division cycle 25 homolog C (S. pombe) |
| CDC42 | cell division cycle 42 (GTP binding protein, 25kDa) |
| CDC42BPG | CDC42 binding protein kinase gamma (DMPK-like) |
| CDC42SE1 | CDC42 small effector 1 |
| CDCA2 | cell division cycle associated 2 |
| CDCA3 | cell division cycle associated 3 |
| CDCA4 | cell division cycle associated 4 |
| CDCA8 | cell division cycle associated 8 |
| CDH24 | cadherin 24, type 2 |
| CDK1 | cyclin-dependent kinase 1 |
| CDK16 | cyclin-dependent kinase 16 |
| CDK20 | cyclin-dependent kinase 20 |
| CDK5R1 | cyclin-dependent kinase 5, regulatory subunit 1 (p35) |
| CDON | Cdon homolog (mouse) |
| CDS1 | CDP-diacylglycerol synthase (phosphatidate cytidylyltransferase) 1 |
| CDYL2 | chromodomain protein, Y-like 2 |
| CEACAM19 | carcinoembryonic antigen-related cell adhesion molecule 19 |
| CEACAM6 | carcinoembryonic antigen-related cell adhesion molecule 6 (non-specific cross reacting antigen) |
| CELF1 | CUGBP, Elav-like family member 1 |
| CELSR1 | cadherin, EGF LAG seven-pass G-type receptor 1 (flamingo homolog, Drosophila) |
| CELSR2 | cadherin, EGF LAG seven-pass G-type receptor 2 (flamingo homolog, Drosophila) |
| CENPJ | centromere protein J |
| CENPM | centromere protein M |
| CENPN | centromere protein N |
| CENPP | centromere protein P |
| CEP152 | centrosomal protein 152kDa |
| CEP250 | centrosomal protein 250kDa |
| CFB | complement factor B |
| CGB7 | chorionic gonadotropin, beta polypeptide 7 |
| CGN | cingulin |
| CHADL | chondroadherin-like |
| CHAT | choline O-acetyltransferase |
| CHEK1 | CHK1 checkpoint homolog (S. pombe) |
| CHFR | checkpoint with forkhead and ring finger domains |
| CHMP4C | chromatin modifying protein 4C |
| CHRD | chordin |
| CHRNA4 | cholinergic receptor, nicotinic, alpha 4 |
| CHRNE | cholinergic receptor, nicotinic, epsilon |
| CHST10 | carbohydrate sulfotransferase 10 |
| CIB1 | calcium and integrin binding 1 (calmyrin) |
| CILP2 | cartilage intermediate layer protein 2 |
| CLCN2 | chloride channel 2 |
| CLCN3 | chloride channel 3 |
| CLDN3 | claudin 3 |
| CLDN7 | claudin 7 |
| CLDND1 | claudin domain containing 1 |
| CLEC5A | C-type lectin domain family 5, member A |
| CLN6 | ceroid-lipofuscinosis, neuronal 6, late infantile, variant |
| CLSPN | claspin homolog (Xenopus laevis) |
| CLSTN1 | calsyntenin 1 |
| CLSTN3 | calsyntenin 3 |
| CNOT6 | CCR4-NOT transcription complex, subunit 6 |
| CNTNAP1 | contactin associated protein 1 |
| COL10A1 | collagen, type X, alpha 1 |
| COL11A1 | collagen, type XI, alpha 1 |
| COL1A1 | collagen, type I, alpha 1 |
| COL1A2 | collagen, type I, alpha 2 |
| COMMD4 | COMM domain containing 4 |
| COMP | cartilage oligomeric matrix protein |
| COQ4 | coenzyme Q4 homolog (S. cerevisiae) |
| COX6C | cytochrome c oxidase subunit VIc |
| COX7B | cytochrome c oxidase subunit VIIb |
| COX7C | cytochrome c oxidase subunit VIIc |
| CPD | carboxypeptidase D |
| CRIP1 | cysteine-rich protein 1 (intestinal) |
| CRNKL1 | crooked neck pre-mRNA splicing factor-like 1 (Drosophila) |
| CRTC1 | CREB regulated transcription coactivator 1 |
| CSH1 | chorionic somatomammotropin hormone 1 (placental lactogen) |
| CSNK1D | casein kinase 1, delta |
| CSNK2A1 | casein kinase 2, alpha 1 polypeptide |
| CTPS | CTP synthase |
| CTXN1 | cortexin 1 |
| CUEDC1 | CUE domain containing 1 |
| CXorf1 | chromosome X open reading frame 1 |
| CYB561 | cytochrome b-561 |
| CYFIP2 | cytoplasmic FMR1 interacting protein 2 |
| DAD1 | defender against cell death 1 |
| DAZAP1 | DAZ associated protein 1 |
| DBNDD2 | dysbindin (dystrobrevin binding protein 1) domain containing 2 |
| DEPDC5 | DEP domain containing 5 |
| DGCR14 | DiGeorge syndrome critical region gene 14 |
| DGKD | diacylglycerol kinase, delta 130kDa |
| DGKE | diacylglycerol kinase, epsilon 64kDa |
| DHX8 | DEAH (Asp-Glu-Ala-His) box polypeptide 8 |
| DHX9 | DEAH (Asp-Glu-Ala-His) box polypeptide 9 |
| DIAPH1 | diaphanous homolog 1 (Drosophila) |
| DIRAS2 | DIRAS family, GTP-binding RAS-like 2 |
| DKFZp761E198 | DKFZp761E198 protein |
| DKFZp761P0212 | hypothetical protein DKFZp761P0212 |
| DLG3 | discs, large homolog 3 (Drosophila) |
| DLGAP5 | discs, large (Drosophila) homolog-associated protein 5 |
| DNA2 | DNA replication helicase 2 homolog (yeast) |
| DNAJA4 | DnaJ (Hsp40) homolog, subfamily A, member 4 |
| DNAJB12 | DnaJ (Hsp40) homolog, subfamily B, member 12 |
| DNASE1L2 | deoxyribonuclease I-like 2 |
| DNM2 | dynamin 2 |
| DOLK | dolichol kinase |
| DONSON | downstream neighbor of SON |
| DPM2 | dolichyl-phosphate mannosyltransferase polypeptide 2, regulatory subunit |
| DPP7 | dipeptidyl-peptidase 7 |
| DUSP15 | dual specificity phosphatase 15 |
| DUSP16 | dual specificity phosphatase 16 |
| DYNLL1 | dynein, light chain, LC8-type 1 |
| DYNLRB1 | dynein, light chain, roadblock-type 1 |
| E2F8 | E2F transcription factor 8 |
| E4F1 | E4F transcription factor 1 |
| EAF1 | ELL associated factor 1 |
| EBNA1BP2 | EBNA1 binding protein 2 |
| ECD | ecdysoneless homolog (Drosophila) |
| EFNA1 | ephrin-A1 |
| EGLN3 | egl nine homolog 3 (C. elegans) |
| EIF1AD | eukaryotic translation initiation factor 1A domain containing |
| EIF2AK1 | eukaryotic translation initiation factor 2-alpha kinase 1 |
| EIF2B4 | eukaryotic translation initiation factor 2B, subunit 4 delta, 67kDa |
| EIF2S3 | eukaryotic translation initiation factor 2, subunit 3 gamma, 52kDa |
| ELAVL1 | ELAV (embryonic lethal, abnormal vision, Drosophila)-like 1 (Hu antigen R) |
| ELF3 | E74-like factor 3 (ets domain transcription factor, epithelial-specific ) |
| ELMOD2 | ELMO/CED-12 domain containing 2 |
| EML2 | echinoderm microtubule associated protein like 2 |
| ENPP1 | ectonucleotide pyrophosphatase/phosphodiesterase 1 |
| ENTPD7 | ectonucleoside triphosphate diphosphohydrolase 7 |
| EPB41 | erythrocyte membrane protein band 4.1 (elliptocytosis 1, RH-linked) |
| EPCAM | epithelial cell adhesion molecule |
| EPN3 | epsin 3 |
| EPR1 | effector cell peptidase receptor 1 (non-protein coding) |
| ERBB3 | v-erb-b2 erythroblastic leukemia viral oncogene homolog 3 (avian) |
| ERBB4 | v-erb-a erythroblastic leukemia viral oncogene homolog 4 (avian) |
| ERF | Ets2 repressor factor |
| ERMN | ermin, ERM-like protein |
| ERVFRDE1 | HERV-FRD provirus ancestral Env polyprotein |
| ESCO2 | establishment of cohesion 1 homolog 2 (S. cerevisiae) |
| ESRP1 | epithelial splicing regulatory protein 1 |
| EVI5L | ecotropic viral integration site 5-like |
| EVL | Enah/Vasp-like |
| EVX1 | even-skipped homeobox 1 |
| EXOC3L | exocyst complex component 3-like |
| EXOC6 | exocyst complex component 6 |
| FAAH | fatty acid amide hydrolase |
| FADS6 | fatty acid desaturase domain family, member 6 |
| FAM104B | family with sequence similarity 104, member B |
| FAM120A | family with sequence similarity 120A |
| FAM128A | family with sequence similarity 128, member A |
| FAM128B | family with sequence similarity 128, member B |
| FAM131A | family with sequence similarity 131, member A |
| FAM131C | family with sequence similarity 131, member C |
| FAM132B | family with sequence similarity 132, member B |
| FAM167A | family with sequence similarity 167, member A |
| FAM169A | family with sequence similarity 169, member A |
| FAM195A | family with sequence similarity 195, member A |
| FAM199X | family with sequence similarity 199, X-linked |
| FAM38B | family with sequence similarity 38, member B |
| FAM43B | family with sequence similarity 43, member B |
| FAM53B | family with sequence similarity 53, member B |
| FAM54A | family with sequence similarity 54, member A |
| FAM64A | family with sequence similarity 64, member A |
| FAM89B | family with sequence similarity 89, member B |
| FANCB | Fanconi anemia, complementation group B |
| FASTKD5 | FAST kinase domains 5 |
| FAT3 | FAT tumor suppressor homolog 3 (Drosophila) |
| FBXL16 | F-box and leucine-rich repeat protein 16 |
| FBXL2 | F-box and leucine-rich repeat protein 2 |
| FBXO17 | F-box protein 17 |
| FBXO41 | F-box protein 41 |
| FBXO6 | F-box protein 6 |
| FCGR1A | Fc fragment of IgG, high affinity Ia, receptor (CD64) |
| FCRL4 | Fc receptor-like 4 |
| FEN1 | flap structure-specific endonuclease 1 |
| FGA | fibrinogen alpha chain |
| FKBP10 | FK506 binding protein 10, 65 kDa |
| FKBP1A | FK506 binding protein 1A, 12kDa |
| FKBP4 | FK506 binding protein 4, 59kDa |
| FLAD1 | FAD1 flavin adenine dinucleotide synthetase homolog (S. cerevisiae) |
| FLJ33360 | FLJ33360 protein |
| FLJ40504 | keratin 18 pseudogene |
| FMR1 | fragile X mental retardation 1 |
| FN3K | fructosamine 3 kinase |
| FOXA1 | forkhead box A1 |
| FPGS | folylpolyglutamate synthase |
| FRAS1 | Fraser syndrome 1 |
| FUT2 | fucosyltransferase 2 (secretor status included) |
| FXYD3 | FXYD domain containing ion transport regulator 3 |
| GAFA1 | FGF2-associated protein GAFA1 |
| GAK | cyclin G associated kinase |
| GAPVD1 | GTPase activating protein and VPS9 domains 1 |
| GATA3 | GATA binding protein 3 |
| GCM1 | glial cells missing homolog 1 (Drosophila) |
| GDAP2 | ganglioside induced differentiation associated protein 2 |
| GEMIN8 | gem (nuclear organelle) associated protein 8 |
| GEMIN8P4 | gem (nuclear organelle) associated protein 8 pseudogene 4 |
| GFOD2 | glucose-fructose oxidoreductase domain containing 2 |
| GGCX | gamma-glutamyl carboxylase |
| GINS1 | GINS complex subunit 1 (Psf1 homolog) |
| GINS2 | GINS complex subunit 2 (Psf2 homolog) |
| GIPC1 | GIPC PDZ domain containing family, member 1 |
| GIPR | gastric inhibitory polypeptide receptor |
| GJB2 | gap junction protein, beta 2, 26kDa |
| GLB1L2 | galactosidase, beta 1-like 2 |
| GLTPD1 | glycolipid transfer protein domain containing 1 |
| GLYR1 | glyoxylate reductase 1 homolog (Arabidopsis) |
| GNAZ | guanine nucleotide binding protein (G protein), alpha z polypeptide |
| GORASP2 | golgi reassembly stacking protein 2, 55kDa |
| GOSR2 | golgi SNAP receptor complex member 2 |
| GPC4 | glypican 4 |
| GPRIN1 | G protein regulated inducer of neurite outgrowth 1 |
| GPSM1 | G-protein signaling modulator 1 (AGS3-like, C. elegans) |
| GPX5 | glutathione peroxidase 5 (epididymal androgen-related protein) |
| GRB7 | growth factor receptor-bound protein 7 |
| GRHL2 | grainyhead-like 2 (Drosophila) |
| GRIPAP1 | GRIP1 associated protein 1 |
| GRLF1 | glucocorticoid receptor DNA binding factor 1 |
| GSR | glutathione reductase |
| GSTO2 | glutathione S-transferase omega 2 |
| GSX1 | GS homeobox 1 |
| GTF2A2 | general transcription factor IIA, 2, 12kDa |
| GTPBP1 | GTP binding protein 1 |
| GTSE1 | G-2 and S-phase expressed 1 |
| H2AFV | H2A histone family, member V |
| H2AFY | H2A histone family, member Y |
| HAUS7 | HAUS augmin-like complex, subunit 7 |
| HAUS8 | HAUS augmin-like complex, subunit 8 |
| HDAC9 | histone deacetylase 9 |
| HEATR7A | HEAT repeat containing 7A |
| HES2 | hairy and enhancer of split 2 (Drosophila) |
| HGS | hepatocyte growth factor-regulated tyrosine kinase substrate |
| HHAT | hedgehog acyltransferase |
| HIST1H1A | histone cluster 1, H1a |
| HIST1H2AB | histone cluster 1, H2ab |
| HIST1H2AC | histone cluster 1, H2ac |
| HIST1H2AD | histone cluster 1, H2ad |
| HIST1H2AE | histone cluster 1, H2ae |
| HIST1H2AI | histone cluster 1, H2ai |
| HIST1H2AJ | histone cluster 1, H2aj |
| HIST1H2AM | histone cluster 1, H2am |
| HIST1H2BG | histone cluster 1, H2bg |
| HIST1H2BK | histone cluster 1, H2bk |
| HIST1H4F | histone cluster 1, H4f |
| HIST1H4H | histone cluster 1, H4h |
| HIST1H4I | histone cluster 1, H4i |
| HIST1H4K | histone cluster 1, H4k |
| HIST2H2AA3 | histone cluster 2, H2aa3 |
| HIST2H2AA4 | histone cluster 2, H2aa4 |
| HIST2H2BE | histone cluster 2, H2be |
| HIST2H4A | histone cluster 2, H4a |
| HIST2H4B | histone cluster 2, H4b |
| HIST3H2A | histone cluster 3, H2a |
| HIVEP3 | human immunodeficiency virus type I enhancer binding protein 3 |
| HJURP | Holliday junction recognition protein |
| HMGN1 | high-mobility group nucleosome binding domain 1 |
| HMGN2 | high-mobility group nucleosomal binding domain 2 |
| HMMR | hyaluronan-mediated motility receptor (RHAMM) |
| HN1L | hematological and neurological expressed 1-like |
| HNRNPAB | heterogeneous nuclear ribonucleoprotein A/B |
| HOXA11 | homeobox A11 |
| HPN | hepsin |
| HS1BP3 | HCLS1 binding protein 3 |
| HSBP1L1 | heat shock factor binding protein 1-like 1 |
| HSD17B1 | hydroxysteroid (17-beta) dehydrogenase 1 |
| HTR3D | 5-hydroxytryptamine (serotonin) receptor 3 family member D |
| HTR3E | 5-hydroxytryptamine (serotonin) receptor 3, family member E |
| HTRA3 | HtrA serine peptidase 3 |
| HUS1 | HUS1 checkpoint homolog (S. pombe) |
| ICK | intestinal cell (MAK-like) kinase |
| IFI30 | interferon, gamma-inducible protein 30 |
| IFT20 | intraflagellar transport 20 homolog (Chlamydomonas) |
| IGHG1 | immunoglobulin heavy constant gamma 1 (G1m marker) |
| IGHG4 | immunoglobulin heavy constant gamma 4 (G4m marker) |
| IGHM | immunoglobulin heavy constant mu |
| IGHV4-31 | immunoglobulin heavy variable 4-31 |
| IGSF22 | immunoglobulin superfamily, member 22 |
| IGSF3 | immunoglobulin superfamily, member 3 |
| IKBKB | inhibitor of kappa light polypeptide gene enhancer in B-cells, kinase beta |
| IL4I1 | interleukin 4 induced 1 |
| IMPAD1 | inositol monophosphatase domain containing 1 |
| INHBC | inhibin, beta C |
| IPO7 | importin 7 |
| IPPK | inositol 1,3,4,5,6-pentakisphosphate 2-kinase |
| IQCD | IQ motif containing D |
| IREB2 | iron-responsive element binding protein 2 |
| IRF6 | interferon regulatory factor 6 |
| IRF7 | interferon regulatory factor 7 |
| ISG20 | interferon stimulated exonuclease gene 20kDa |
| ITGB6 | integrin, beta 6 |
| ITPA | inosine triphosphatase (nucleoside triphosphate pyrophosphatase) |
| JMJD7 | jumonji domain containing 7 |
| JMJD7-PLA2G4B | JMJD7-PLA2G4B readthrough |
| JOSD2 | Josephin domain containing 2 |
| JPH1 | junctophilin 1 |
| JRK | jerky homolog (mouse) |
| JUP | junction plakoglobin |
| KAL1 | Kallmann syndrome 1 sequence |
| KCNJ1 | potassium inwardly-rectifying channel, subfamily J, member 1 |
| KCNK6 | potassium channel, subfamily K, member 6 |
| KDELR2 | KDEL (Lys-Asp-Glu-Leu) endoplasmic reticulum protein retention receptor 2 |
| KDM4B | lysine (K)-specific demethylase 4B |
| KIAA0101 | KIAA0101 |
| KIAA0317 | KIAA0317 |
| KIAA0556 | KIAA0556 |
| KIAA0562 | KIAA0562 |
| KIAA1244 | KIAA1244 |
| KIAA1324 | KIAA1324 |
| KIAA1468 | KIAA1468 |
| KIAA1841 | KIAA1841 |
| KIF11 | kinesin family member 11 |
| KIF16B | kinesin family member 16B |
| KIF18B | kinesin family member 18B |
| KIF20A | kinesin family member 20A |
| KIF26B | kinesin family member 26B |
| KIF2C | kinesin family member 2C |
| KIFC1 | kinesin family member C1 |
| KIR2DL4 | killer cell immunoglobulin-like receptor, two domains, long cytoplasmic tail, 4 |
| KIRREL | kin of IRRE like (Drosophila) |
| KISS1 | KiSS-1 metastasis-suppressor |
| KLC2 | kinesin light chain 2 |
| KLHDC10 | kelch domain containing 10 |
| KLHDC5 | kelch domain containing 5 |
| KNTC1 | kinetochore associated 1 |
| KRT18 | keratin 18 |
| KRT19 | keratin 19 |
| KRT19P2 | keratin 19 pseudogene 2 |
| KRT76 | keratin 76 |
| KRT8 | keratin 8 |
| KRT8P12 | keratin 8 pseudogene 12 |
| KRTCAP3 | keratinocyte associated protein 3 |
| LARP1 | La ribonucleoprotein domain family, member 1 |
| LASS3 | LAG1 homolog, ceramide synthase 3 |
| LBH | limb bud and heart development homolog (mouse) |
| LCMT2 | leucine carboxyl methyltransferase 2 |
| LCOR | ligand dependent nuclear receptor corepressor |
| LETM1 | leucine zipper-EF-hand containing transmembrane protein 1 |
| LGALS8 | lectin, galactoside-binding, soluble, 8 |
| LGALS9 | lectin, galactoside-binding, soluble, 9 |
| LGALS9C | lectin, galactoside-binding, soluble, 9C |
| LHFPL5 | lipoma HMGIC fusion partner-like 5 |
| LIG1 | ligase I, DNA, ATP-dependent |
| LIG3 | ligase III, DNA, ATP-dependent |
| LIMD2 | LIM domain containing 2 |
| LIMK1 | LIM domain kinase 1 |
| LIN28B | lin-28 homolog B (C. elegans) |
| LLGL2 | lethal giant larvae homolog 2 (Drosophila) |
| LOC100127913 | hypothetical LOC100127913 |
| LOC100128401 | hypothetical protein LOC100128401 |
| LOC100128844 | hypothetical protein LOC100128844 |
| LOC100129455 | hypothetical LOC100129455 |
| LOC100129463 | hypothetical LOC100129463 |
| LOC100129775 | hypothetical protein LOC100129775 |
| LOC100129857 | hypothetical LOC100129857 |
| LOC100129917 | hypothetical protein LOC100129917 |
| LOC100130776 | hypothetical LOC100130776 |
| LOC100130930 | hypothetical LOC100130930 |
| LOC100131091 | hypothetical LOC100131091 |
| LOC100132779 | hypothetical LOC100132779 |
| LOC100289143 | similar to nucleolar protein family A, member 3 |
| LOC147804 | tropomyosin 3 pseudogene |
| LOC149501 | similar to keratin 8 |
| LOC221442 | adenylate cyclase 10 pseudogene |
| LOC284889 | hypothetical protein LOC284889 |
| LOC338579 | hypothetical protein LOC338579 |
| LOC338667 | hypothetical protein LOC338667 |
| LOC377711 | KIAA1833-like |
| LOC400099 | hypothetical LOC400099 |
| LOC440895 | LIM and senescent cell antigen-like domains 3-like |
| LOC441155 | zinc finger CCCH-type domain-containing pseudogene |
| LOC441455 | makorin ring finger protein 1 pseudogene |
| LOC442249 | similar to keratin 18 |
| LOC642852 | hypothetical LOC642852 |
| LOC643896 | cAMP-regulated phosphoprotein 19 pseudogene |
| LOC644422 | similar to arginine/serine-rich splicing factor 6 |
| LOC645181 | similar to PDGFA associated protein 1 |
| LOC645405 | similar to oxidation resistance 1 |
| LOC647859 | occludin pseudogene |
| LOC653348 | similar to ADAMTS-like 2 |
| LOC653888 | similar to Actin-related protein 2/3 complex subunit 1B (ARP2/3 complex 41 kDa subunit) (p41-ARC) |
| LOC728003 | similar to vesicle-associated membrane protein-associated protein A |
| LOC732419 | similar to AAA-ATPase TOB3 |
| LOC91948 | hypothetical LOC91948 |
| LOC92659 | hypothetical LOC92659 |
| LONRF2 | LON peptidase N-terminal domain and ring finger 2 |
| LOR | loricrin |
| LRBA | LPS-responsive vesicle trafficking, beach and anchor containing |
| LRRC15 | leucine rich repeat containing 15 |
| LRRC4 | leucine rich repeat containing 4 |
| LRRC45 | leucine rich repeat containing 45 |
| LRRC50 | leucine rich repeat containing 50 |
| LRRC56 | leucine rich repeat containing 56 |
| LRRC59 | leucine rich repeat containing 59 |
| LRRC68 | leucine rich repeat containing 68 |
| LRRC8E | leucine rich repeat containing 8 family, member E |
| LRRIQ3 | leucine-rich repeats and IQ motif containing 3 |
| LRRN2 | leucine rich repeat neuronal 2 |
| LRRTM1 | leucine rich repeat transmembrane neuronal 1 |
| LRTOMT | leucine rich transmembrane and 0-methyltransferase domain containing |
| LYPLA2P1 | lysophospholipase II pseudogene 1 |
| LYSMD1 | LysM, putative peptidoglycan-binding, domain containing 1 |
| MAGI3 | membrane associated guanylate kinase, WW and PDZ domain containing 3 |
| MAGOHB | mago-nashi homolog B (Drosophila) |
| MANF | mesencephalic astrocyte-derived neurotrophic factor |
| MAP1S | microtubule-associated protein 1S |
| MAP3K2 | mitogen-activated protein kinase kinase kinase 2 |
| MAPT | microtubule-associated protein tau |
| MARVELD2 | MARVEL domain containing 2 |
| MAS1L | MAS1 oncogene-like |
| MATN3 | matrilin 3 |
| MAZ | MYC-associated zinc finger protein (purine-binding transcription factor) |
| MCM5 | minichromosome maintenance complex component 5 |
| MCM8 | minichromosome maintenance complex component 8 |
| MEAF6 | MYST/Esa1-associated factor 6 |
| MED29 | mediator complex subunit 29 |
| MELK | maternal embryonic leucine zipper kinase |
| MFSD3 | major facilitator superfamily domain containing 3 |
| MICALL2 | MICAL-like 2 |
| MIF | macrophage migration inhibitory factor (glycosylation-inhibiting factor) |
| MKI67 | antigen identified by monoclonal antibody Ki-67 |
| MKL2 | MKL/myocardin-like 2 |
| MLEC | malectin |
| MLL2 | myeloid/lymphoid or mixed-lineage leukemia 2 |
| MLL3 | myeloid/lymphoid or mixed-lineage leukemia 3 |
| MLLT10 | myeloid/lymphoid or mixed-lineage leukemia (trithorax homolog, Drosophila); translocated to, 10 |
| MLPH | melanophilin |
| MMP14 | matrix metallopeptidase 14 (membrane-inserted) |
| MMP9 | matrix metallopeptidase 9 (gelatinase B, 92kDa gelatinase, 92kDa type IV collagenase) |
| MPPE1 | metallophosphoesterase 1 |
| MRPL30 | mitochondrial ribosomal protein L30 |
| MRPL47 | mitochondrial ribosomal protein L47 |
| MRPS34 | mitochondrial ribosomal protein S34 |
| MRPS36 | mitochondrial ribosomal protein S36 |
| MS4A14 | membrane-spanning 4-domains, subfamily A, member 14 |
| MSI2 | musashi homolog 2 (Drosophila) |
| MTHFR | methylenetetrahydrofolate reductase (NAD(P)H) |
| MTP18 | mitochondrial protein 18 kDa |
| MUC1 | mucin 1, cell surface associated |
| MYBL1 | v-myb myeloblastosis viral oncogene homolog (avian)-like 1 |
| MYEF2 | myelin expression factor 2 |
| MYL12B | myosin, light chain 12B, regulatory |
| MYO1B | myosin IB |
| MYO1D | myosin ID |
| MYO5A | myosin VA (heavy chain 12, myoxin) |
| MYOF | myoferlin |
| MYST3 | MYST histone acetyltransferase (monocytic leukemia) 3 |
| N4BP2 | NEDD4 binding protein 2 |
| NACC1 | nucleus accumbens associated 1, BEN and BTB (POZ) domain containing |
| NADSYN1 | NAD synthetase 1 |
| NCAPH | non-SMC condensin I complex, subunit H |
| NCAPH2 | non-SMC condensin II complex, subunit H2 |
| NCBP1 | nuclear cap binding protein subunit 1, 80kDa |
| NCOA5 | nuclear receptor coactivator 5 |
| NCRNA00205 | non-protein coding RNA 205 |
| NDC80 | NDC80 homolog, kinetochore complex component (S. cerevisiae) |
| NDFIP1 | Nedd4 family interacting protein 1 |
| NDNL2 | necdin-like 2 |
| NDUFA1 | NADH dehydrogenase (ubiquinone) 1 alpha subcomplex, 1, 7.5kDa |
| NDUFA7 | NADH dehydrogenase (ubiquinone) 1 alpha subcomplex, 7, 14.5kDa |
| NEDD8 | neural precursor cell expressed, developmentally down-regulated 8 |
| NEK6 | NIMA (never in mitosis gene a)-related kinase 6 |
| NF1 | neurofibromin 1 |
| NFATC2IP | nuclear factor of activated T-cells, cytoplasmic, calcineurin-dependent 2 interacting protein |
| NFKBIE | nuclear factor of kappa light polypeptide gene enhancer in B-cells inhibitor, epsilon |
| NGRN | neugrin, neurite outgrowth associated |
| NHLH2 | nescient helix loop helix 2 |
| NKD1 | naked cuticle homolog 1 (Drosophila) |
| NME3 | non-metastatic cells 3, protein expressed in |
| NME5 | non-metastatic cells 5, protein expressed in (nucleoside-diphosphate kinase) |
| NOP10 | NOP10 ribonucleoprotein homolog (yeast) |
| NUFIP2 | nuclear fragile X mental retardation protein interacting protein 2 |
| NUP210 | nucleoporin 210kDa |
| OAS2 | 2'-5'-oligoadenylate synthetase 2, 69/71kDa |
| OCLN | occludin |
| OLR1 | oxidized low density lipoprotein (lectin-like) receptor 1 |
| OPA3 | optic atrophy 3 (autosomal recessive, with chorea and spastic paraplegia) |
| OR13F1 | olfactory receptor, family 13, subfamily F, member 1 |
| OR1L6 | olfactory receptor, family 1, subfamily L, member 6 |
| OR2T11 | olfactory receptor, family 2, subfamily T, member 11 |
| OR4C15 | olfactory receptor, family 4, subfamily C, member 15 |
| OR4C16 | olfactory receptor, family 4, subfamily C, member 16 |
| OR4K17 | olfactory receptor, family 4, subfamily K, member 17 |
| OR4M2 | olfactory receptor, family 4, subfamily M, member 2 |
| OR4X2 | olfactory receptor, family 4, subfamily X, member 2 |
| OR51M1 | olfactory receptor, family 51, subfamily M, member 1 |
| OR5AK2 | olfactory receptor, family 5, subfamily AK, member 2 |
| OR5D16 | olfactory receptor, family 5, subfamily D, member 16 |
| OR5F1 | olfactory receptor, family 5, subfamily F, member 1 |
| ORC1L | origin recognition complex, subunit 1-like (yeast) |
| OSCP1 | organic solute carrier partner 1 |
| OTOS | otospiralin |
| OVOL1 | ovo-like 1(Drosophila) |
| OVOL2 | ovo-like 2 (Drosophila) |
| OXR1 | oxidation resistance 1 |
| P2RY2 | purinergic receptor P2Y, G-protein coupled, 2 |
| PAK4 | p21 protein (Cdc42/Rac)-activated kinase 4 |
| PANK3 | pantothenate kinase 3 |
| PAPPA2 | pappalysin 2 |
| PAQR4 | progestin and adipoQ receptor family member IV |
| PARP10 | poly (ADP-ribose) polymerase family, member 10 |
| PARS2 | prolyl-tRNA synthetase 2, mitochondrial (putative) |
| PASD1 | PAS domain containing 1 |
| PBK | PDZ binding kinase |
| PBX2 | pre-B-cell leukemia homeobox 2 |
| PCDHGA8 | protocadherin gamma subfamily A, 8 |
| PCDHGB4 | protocadherin gamma subfamily B, 4 |
| PCLO | piccolo (presynaptic cytomatrix protein) |
| PCNXL3 | pecanex-like 3 (Drosophila) |
| PDAP1 | PDGFA associated protein 1 |
| PDZD7 | PDZ domain containing 7 |
| PFKFB2 | 6-phosphofructo-2-kinase/fructose-2,6-biphosphatase 2 |
| PGAM5 | phosphoglycerate mutase family member 5 |
| PGLYRP3 | peptidoglycan recognition protein 3 |
| PHF15 | PHD finger protein 15 |
| PHF2 | PHD finger protein 2 |
| PHF20L1 | PHD finger protein 20-like 1 |
| PHF6 | PHD finger protein 6 |
| PHF8 | PHD finger protein 8 |
| PHLDB3 | pleckstrin homology-like domain, family B, member 3 |
| PHTF2 | putative homeodomain transcription factor 2 |
| PHYH | phytanoyl-CoA 2-hydroxylase |
| PI4K2A | phosphatidylinositol 4-kinase type 2 alpha |
| PIGT | phosphatidylinositol glycan anchor biosynthesis, class T |
| PIP5K1A | phosphatidylinositol-4-phosphate 5-kinase, type I, alpha |
| PITX3 | paired-like homeodomain 3 |
| PKIB | protein kinase (cAMP-dependent, catalytic) inhibitor beta |
| PKM2 | pyruvate kinase, muscle |
| PKMYT1 | protein kinase, membrane associated tyrosine/threonine 1 |
| PKNOX1 | PBX/knotted 1 homeobox 1 |
| PLA2G10 | phospholipase A2, group X |
| PLA2G12A | phospholipase A2, group XIIA |
| PLEKHB2 | pleckstrin homology domain containing, family B (evectins) member 2 |
| PLK1 | polo-like kinase 1 (Drosophila) |
| PLP2 | proteolipid protein 2 (colonic epithelium-enriched) |
| PNKP | polynucleotide kinase 3'-phosphatase |
| PNPLA5 | patatin-like phospholipase domain containing 5 |
| POC1A | POC1 centriolar protein homolog A (Chlamydomonas) |
| POGK | pogo transposable element with KRAB domain |
| POLA1 | polymerase (DNA directed), alpha 1, catalytic subunit |
| POLD1 | polymerase (DNA directed), delta 1, catalytic subunit 125kDa |
| POLM | polymerase (DNA directed), mu |
| POLQ | polymerase (DNA directed), theta |
| POLR2J3 | polymerase (RNA) II (DNA directed) polypeptide J3 |
| POLR2J4 | polymerase (RNA) II (DNA directed) polypeptide J4, pseudogene |
| POLR3A | polymerase (RNA) III (DNA directed) polypeptide A, 155kDa |
| POMP | proteasome maturation protein |
| POP1 | processing of precursor 1, ribonuclease P/MRP subunit (S. cerevisiae) |
| POTED | POTE ankyrin domain family, member D |
| PPAP2C | phosphatidic acid phosphatase type 2C |
| PPCDC | phosphopantothenoylcysteine decarboxylase |
| PPM1E | protein phosphatase, Mg2+/Mn2+ dependent, 1E |
| PPM1H | protein phosphatase, Mg2+/Mn2+ dependent, 1H |
| PPP1CA | protein phosphatase 1, catalytic subunit, alpha isozyme |
| PPP4C | protein phosphatase 4, catalytic subunit |
| PQLC2 | PQ loop repeat containing 2 |
| PRAF2 | PRA1 domain family, member 2 |
| PRICKLE3 | prickle homolog 3 (Drosophila) |
| PRKCD | protein kinase C, delta |
| PRLR | prolactin receptor |
| PROP1 | PROP paired-like homeobox 1 |
| PRR11 | proline rich 11 |
| PRR15 | proline rich 15 |
| PRR21 | proline rich 21 |
| PRSS1 | protease, serine, 1 (trypsin 1) |
| PRSS2 | protease, serine, 2 (trypsin 2) |
| PRSS8 | protease, serine, 8 |
| PSMA5 | proteasome (prosome, macropain) subunit, alpha type, 5 |
| PSMA6 | proteasome (prosome, macropain) subunit, alpha type, 6 |
| PSMA7 | proteasome (prosome, macropain) subunit, alpha type, 7 |
| PSPN | persephin |
| PTDSS1 | phosphatidylserine synthase 1 |
| PTGES2 | prostaglandin E synthase 2 |
| PTPRT | protein tyrosine phosphatase, receptor type, T |
| PTTG1 | pituitary tumor-transforming 1 |
| PTTG2 | pituitary tumor-transforming 2 |
| PTTG3P | pituitary tumor-transforming 3 (pseudogene) |
| PVRL2 | poliovirus receptor-related 2 (herpesvirus entry mediator B) |
| PWP2 | PWP2 periodic tryptophan protein homolog (yeast) |
| PYCR1 | pyrroline-5-carboxylate reductase 1 |
| PYCRL | pyrroline-5-carboxylate reductase-like |
| QRICH1 | glutamine-rich 1 |
| R3HDM1 | R3H domain containing 1 |
| RAB11FIP3 | RAB11 family interacting protein 3 (class II) |
| RAB17 | RAB17, member RAS oncogene family |
| RAB26 | RAB26, member RAS oncogene family |
| RAB3D | RAB3D, member RAS oncogene family |
| RAB40C | RAB40C, member RAS oncogene family |
| RABEP2 | rabaptin, RAB GTPase binding effector protein 2 |
| RABL5 | RAB, member RAS oncogene family-like 5 |
| RAC1 | ras-related C3 botulinum toxin substrate 1 (rho family, small GTP binding protein Rac1) |
| RAD9A | RAD9 homolog A (S. pombe) |
| RASEF | RAS and EF-hand domain containing |
| RBAK | RB-associated KRAB zinc finger |
| RBM33 | RNA binding motif protein 33 |
| RCC2 | regulator of chromosome condensation 2 |
| RDH12 | retinol dehydrogenase 12 (all-trans/9-cis/11-cis) |
| RELT | RELT tumor necrosis factor receptor |
| RER1 | RER1 retention in endoplasmic reticulum 1 homolog (S. cerevisiae) |
| RFK | riboflavin kinase |
| RFX1 | regulatory factor X, 1 (influences HLA class II expression) |
| RFXANK | regulatory factor X-associated ankyrin-containing protein |
| RG9MTD2 | RNA (guanine-9-) methyltransferase domain containing 2 |
| RGS16 | regulator of G-protein signaling 16 |
| RGS9BP | regulator of G protein signaling 9 binding protein |
| RHBDD3 | rhomboid domain containing 3 |
| RHCE | Rh blood group, CcEe antigens |
| RHOC | ras homolog gene family, member C |
| RHOT1 | ras homolog gene family, member T1 |
| RHOT2 | ras homolog gene family, member T2 |
| RHPN2P1 | rhophilin, Rho GTPase binding protein 2 pseudogene 1 |
| RNASE8 | ribonuclease, RNase A family, 8 |
| RNF123 | ring finger protein 123 |
| RNF149 | ring finger protein 149 |
| RNF151 | ring finger protein 151 |
| RNF187 | ring finger protein 187 |
| RNF208 | ring finger protein 208 |
| RNF213 | ring finger protein 213 |
| RNF5 | ring finger protein 5 |
| RNF5P1 | ring finger protein 5 pseudogene 1 |
| RNPS1 | RNA binding protein S1, serine-rich domain |
| RORC | RAR-related orphan receptor C |
| RPP14 | ribonuclease P/MRP 14kDa subunit |
| RPRD1B | regulation of nuclear pre-mRNA domain containing 1B |
| RPS6KA1 | ribosomal protein S6 kinase, 90kDa, polypeptide 1 |
| RRM2 | ribonucleotide reductase M2 |
| RSPH6A | radial spoke head 6 homolog A (Chlamydomonas) |
| RTDR1 | rhabdoid tumor deletion region gene 1 |
| RTN4R | reticulon 4 receptor |
| RUNX1 | runt-related transcription factor 1 |
| SAMD1 | sterile alpha motif domain containing 1 |
| SAP18 | Sin3A-associated protein, 18kDa |
| SBK1 | SH3-binding domain kinase 1 |
| SBK2 | SH3-binding domain kinase family, member 2 |
| SCAMP5 | secretory carrier membrane protein 5 |
| SCLY | selenocysteine lyase |
| SCNN1A | sodium channel, nonvoltage-gated 1 alpha |
| SDF4 | stromal cell derived factor 4 |
| SEH1L | SEH1-like (S. cerevisiae) |
| SEMA4G | sema domain, immunoglobulin domain (Ig), transmembrane domain (TM) and short cytoplasmic domain, (semaphorin) 4G |
| SERF1A | small EDRK-rich factor 1A (telomeric) |
| SERF1B | small EDRK-rich factor 1B (centromeric) |
| SERPINA11 | serpin peptidase inhibitor, clade A (alpha-1 antiproteinase, antitrypsin), member 11 |
| SERPINA3 | serpin peptidase inhibitor, clade A (alpha-1 antiproteinase, antitrypsin), member 3 |
| SERPINE1 | serpin peptidase inhibitor, clade E (nexin, plasminogen activator inhibitor type 1), member 1 |
| SETD8 | SET domain containing (lysine methyltransferase) 8 |
| SFI1 | Sfi1 homolog, spindle assembly associated (yeast) |
| SFRS6 | splicing factor, arginine/serine-rich 6 |
| SFT2D3 | SFT2 domain containing 3 |
| SFTPA1 | surfactant protein A1 |
| SFTPA2 | surfactant protein A2 |
| SFXN5 | sideroflexin 5 |
| SGOL1 | shugoshin-like 1 (S. pombe) |
| SH2D3A | SH2 domain containing 3A |
| SH3BGRL3 | SH3 domain binding glutamic acid-rich protein like 3 |
| SH3BP1 | SH3-domain binding protein 1 |
| SH3RF1 | SH3 domain containing ring finger 1 |
| SHC1 | SHC (Src homology 2 domain containing) transforming protein 1 |
| SHC2 | SHC (Src homology 2 domain containing) transforming protein 2 |
| SHKBP1 | SH3KBP1 binding protein 1 |
| SIGLEC7 | sialic acid binding Ig-like lectin 7 |
| SIPA1L3 | signal-induced proliferation-associated 1 like 3 |
| SIRPA | signal-regulatory protein alpha |
| SKA1 | spindle and kinetochore associated complex subunit 1 |
| SKA3 | spindle and kinetochore associated complex subunit 3 |
| SLC19A1 | solute carrier family 19 (folate transporter), member 1 |
| SLC20A1 | solute carrier family 20 (phosphate transporter), member 1 |
| SLC22A10 | solute carrier family 22, member 10 |
| SLC25A10 | solute carrier family 25 (mitochondrial carrier; dicarboxylate transporter), member 10 |
| SLC25A15 | solute carrier family 25 (mitochondrial carrier; ornithine transporter) member 15 |
| SLC25A39 | solute carrier family 25, member 39 |
| SLC26A6 | solute carrier family 26, member 6 |
| SLC2A1 | solute carrier family 2 (facilitated glucose transporter), member 1 |
| SLC2A5 | solute carrier family 2 (facilitated glucose/fructose transporter), member 5 |
| SLC2A6 | solute carrier family 2 (facilitated glucose transporter), member 6 |
| SLC35A2 | solute carrier family 35 (UDP-galactose transporter), member A2 |
| SLC37A1 | solute carrier family 37 (glycerol-3-phosphate transporter), member 1 |
| SLC38A1 | solute carrier family 38, member 1 |
| SLC44A4 | solute carrier family 44, member 4 |
| SLC7A1 | solute carrier family 7 (cationic amino acid transporter, y+ system), member 1 |
| SLC7A4 | solute carrier family 7 (cationic amino acid transporter, y+ system), member 4 |
| SMG7 | Smg-7 homolog, nonsense mediated mRNA decay factor (C. elegans) |
| SMPDL3B | sphingomyelin phosphodiesterase, acid-like 3B |
| SMU1 | smu-1 suppressor of mec-8 and unc-52 homolog (C. elegans) |
| SNAP29 | synaptosomal-associated protein, 29kDa |
| SNRPD3 | small nuclear ribonucleoprotein D3 polypeptide 18kDa |
| SNX12 | sorting nexin 12 |
| SNX19 | sorting nexin 19 |
| SNX8 | sorting nexin 8 |
| SOCS7 | suppressor of cytokine signaling 7 |
| SOLH | small optic lobes homolog (Drosophila) |
| SORCS1 | sortilin-related VPS10 domain containing receptor 1 |
| SP1 | Sp1 transcription factor |
| SPATA13 | spermatogenesis associated 13 |
| SPDEF | SAM pointed domain containing ets transcription factor |
| SPEF1 | sperm flagellar 1 |
| SPG11 | spastic paraplegia 11 (autosomal recessive) |
| SPG21 | spastic paraplegia 21 (autosomal recessive, Mast syndrome) |
| SPINT2 | serine peptidase inhibitor, Kunitz type, 2 |
| SPIRE2 | spire homolog 2 (Drosophila) |
| SPRR1B | small proline-rich protein 1B (cornifin) |
| SQLE | squalene epoxidase |
| SRC | v-src sarcoma (Schmidt-Ruppin A-2) viral oncogene homolog (avian) |
| SRRM2 | serine/arginine repetitive matrix 2 |
| SSBP4 | single stranded DNA binding protein 4 |
| SSH1 | slingshot homolog 1 (Drosophila) |
| SSNA1 | Sjogren syndrome nuclear autoantigen 1 |
| SSR1 | signal sequence receptor, alpha |
| ST14 | suppression of tumorigenicity 14 (colon carcinoma) |
| STAP2 | signal transducing adaptor family member 2 |
| STARD10 | StAR-related lipid transfer (START) domain containing 10 |
| STK39 | serine threonine kinase 39 (STE20/SPS1 homolog, yeast) |
| STOML1 | stomatin (EPB72)-like 1 |
| STRA13 | stimulated by retinoic acid 13 homolog (mouse) |
| STRBP | spermatid perinuclear RNA binding protein |
| STX4 | syntaxin 4 |
| SULT4A1 | sulfotransferase family 4A, member 1 |
| SYCE2 | synaptonemal complex central element protein 2 |
| SYS1-DBNDD2 | SYS1-DBNDD2 readthrough transcript |
| SYT13 | synaptotagmin XIII |
| SYT9 | synaptotagmin IX |
| TACC3 | transforming, acidic coiled-coil containing protein 3 |
| TALDO1 | transaldolase 1 |
| TATDN2 | TatD DNase domain containing 2 |
| TBC1D10B | TBC1 domain family, member 10B |
| TBXA2R | thromboxane A2 receptor |
| TCEA3 | transcription elongation factor A (SII), 3 |
| TCP10L | t-complex 10 (mouse)-like |
| TCP10L2 | t-complex 10-like 2 (mouse) |
| TCTEX1D2 | Tctex1 domain containing 2 |
| TEKT4 | tektin 4 |
| TFAP4 | transcription factor AP-4 (activating enhancer binding protein 4) |
| TFDP1 | transcription factor Dp-1 |
| TGFBI | transforming growth factor, beta-induced, 68kDa |
| TGFBRAP1 | transforming growth factor, beta receptor associated protein 1 |
| THAP8 | THAP domain containing 8 |
| TICAM1 | toll-like receptor adaptor molecule 1 |
| TIMELESS | timeless homolog (Drosophila) |
| TIMM23 | translocase of inner mitochondrial membrane 23 homolog (yeast) |
| TIMP1 | TIMP metallopeptidase inhibitor 1 |
| TK1 | thymidine kinase 1, soluble |
| TMC4 | transmembrane channel-like 4 |
| TMC6 | transmembrane channel-like 6 |
| TMED8 | transmembrane emp24 protein transport domain containing 8 |
| TMEM115 | transmembrane protein 115 |
| TMEM125 | transmembrane protein 125 |
| TMEM222 | transmembrane protein 222 |
| TMEM41A | transmembrane protein 41A |
| TMEM50A | transmembrane protein 50A |
| TMEM63B | transmembrane protein 63B |
| TMEM86A | transmembrane protein 86A |
| TMEM87B | transmembrane protein 87B |
| TMEM89 | transmembrane protein 89 |
| TMUB1 | transmembrane and ubiquitin-like domain containing 1 |
| TNFRSF9 | tumor necrosis factor receptor superfamily, member 9 |
| TNNI3 | troponin I type 3 (cardiac) |
| TNP2 | transition protein 2 (during histone to protamine replacement) |
| TNPO2 | transportin 2 |
| TOP2A | topoisomerase (DNA) II alpha 170kDa |
| TOX3 | TOX high mobility group box family member 3 |
| TPD52 | tumor protein D52 |
| TPD52L3 | tumor protein D52-like 3 |
| TPM3 | tropomyosin 3 |
| TPX2 | TPX2, microtubule-associated, homolog (Xenopus laevis) |
| TRAF7 | TNF receptor-associated factor 7 |
| TRAPPC9 | trafficking protein particle complex 9 |
| TREX2 | three prime repair exonuclease 2 |
| TRIB3 | tribbles homolog 3 (Drosophila) |
| TRIM10 | tripartite motif-containing 10 |
| TRIM14 | tripartite motif-containing 14 |
| TRIM25 | tripartite motif-containing 25 |
| TRIM62 | tripartite motif-containing 62 |
| TRMT2A | TRM2 tRNA methyltransferase 2 homolog A (S. cerevisiae) |
| TROAP | trophinin associated protein (tastin) |
| TSPAN1 | tetraspanin 1 |
| TSPAN17 | tetraspanin 17 |
| TSSC4 | tumor suppressing subtransferable candidate 4 |
| TSSK2 | testis-specific serine kinase 2 |
| TTC26 | tetratricopeptide repeat domain 26 |
| TTC39A | tetratricopeptide repeat domain 39A |
| TTC39C | tetratricopeptide repeat domain 39C |
| TUBA1B | tubulin, alpha 1b |
| TUBA1C | tubulin, alpha 1c |
| TUBB3 | tubulin, beta 3 |
| TUBB4Q | tubulin, beta polypeptide 4, member Q |
| TXN | thioredoxin |
| TYMP | thymidine phosphorylase |
| TYR | tyrosinase (oculocutaneous albinism IA) |
| U2AF2 | U2 small nuclear RNA auxiliary factor 2 |
| UBE2C | ubiquitin-conjugating enzyme E2C |
| UBE2D4 | ubiquitin-conjugating enzyme E2D 4 (putative) |
| UBE2H | ubiquitin-conjugating enzyme E2H (UBC8 homolog, yeast) |
| UBE2K | ubiquitin-conjugating enzyme E2K (UBC1 homolog, yeast) |
| UBE2Z | ubiquitin-conjugating enzyme E2Z |
| UBFD1 | ubiquitin family domain containing 1 |
| UBN1 | ubinuclein 1 |
| UBQLN4 | ubiquilin 4 |
| UBXN10 | UBX domain protein 10 |
| UBXN11 | UBX domain protein 11 |
| UBXN2B | UBX domain protein 2B |
| UBXN6 | UBX domain protein 6 |
| UCN | urocortin |
| UGGT1 | UDP-glucose glycoprotein glucosyltransferase 1 |
| UHRF1 | ubiquitin-like with PHD and ring finger domains 1 |
| UHRF1BP1 | UHRF1 binding protein 1 |
| UNC13A | unc-13 homolog A (C. elegans) |
| UNC45A | unc-45 homolog A (C. elegans) |
| UNC5A | unc-5 homolog A (C. elegans) |
| UNC5B | unc-5 homolog B (C. elegans) |
| UQCR10 | ubiquinol-cytochrome c reductase, complex III subunit X |
| USF2 | upstream transcription factor 2, c-fos interacting |
| USP38 | ubiquitin specific peptidase 38 |
| USP6 | ubiquitin specific peptidase 6 (Tre-2 oncogene) |
| UTF1 | undifferentiated embryonic cell transcription factor 1 |
| VANGL1 | vang-like 1 (van gogh, Drosophila) |
| VAV3 | vav 3 guanine nucleotide exchange factor |
| VDAC1 | voltage-dependent anion channel 1 |
| VEGFA | vascular endothelial growth factor A |
| VGLL2 | vestigial like 2 (Drosophila) |
| VPS53 | vacuolar protein sorting 53 homolog (S. cerevisiae) |
| VWA1 | von Willebrand factor A domain containing 1 |
| WBSCR22 | Williams Beuren syndrome chromosome region 22 |
| WBSCR28 | Williams-Beuren syndrome chromosome region 28 |
| WDR24 | WD repeat domain 24 |
| WDR36 | WD repeat domain 36 |
| WDR55 | WD repeat domain 55 |
| WDR6 | WD repeat domain 6 |
| WDR62 | WD repeat domain 62 |
| WFDC2 | WAP four-disulfide core domain 2 |
| WHSC1 | Wolf-Hirschhorn syndrome candidate 1 |
| WHSC1L1 | Wolf-Hirschhorn syndrome candidate 1-like 1 |
| XBP1 | X-box binding protein 1 |
| XIAP | X-linked inhibitor of apoptosis |
| XPNPEP3 | X-prolyl aminopeptidase (aminopeptidase P) 3, putative |
| XPO6 | exportin 6 |
| XPR1 | xenotropic and polytropic retrovirus receptor 1 |
| YRDC | yrdC domain containing (E. coli) |
| YWHAG | tyrosine 3-monooxygenase/tryptophan 5-monooxygenase activation protein, gamma polypeptide |
| YWHAZ | tyrosine 3-monooxygenase/tryptophan 5-monooxygenase activation protein, zeta polypeptide |
| ZBTB10 | zinc finger and BTB domain containing 10 |
| ZC3H12D | zinc finger CCCH-type containing 12D |
| ZC3H13 | zinc finger CCCH-type containing 13 |
| ZC3H3 | zinc finger CCCH-type containing 3 |
| ZDHHC6 | zinc finger, DHHC-type containing 6 |
| ZKSCAN4 | zinc finger with KRAB and SCAN domains 4 |
| ZKSCAN5 | zinc finger with KRAB and SCAN domains 5 |
| ZMYND10 | zinc finger, MYND-type containing 10 |
| ZNF213 | zinc finger protein 213 |
| ZNF214 | zinc finger protein 214 |
| ZNF24 | zinc finger protein 24 |
| ZNF254 | zinc finger protein 254 |
| ZNF443 | zinc finger protein 443 |
| ZNF444 | zinc finger protein 444 |
| ZNF48 | zinc finger protein 48 |
| ZNF486 | zinc finger protein 486 |
| ZNF500 | zinc finger protein 500 |
| ZNF516 | zinc finger protein 516 |
| ZNF557 | zinc finger protein 557 |
| ZNF576 | zinc finger protein 576 |
| ZNF611 | zinc finger protein 611 |
| ZNF664 | zinc finger protein 664 |
| ZNF668 | zinc finger protein 668 |
| ZNF682 | zinc finger protein 682 |
| ZNF703 | zinc finger protein 703 |
| ZNF764 | zinc finger protein 764 |
| ZNF771 | zinc finger protein 771 |
| ZNF862 | zinc finger protein 862 |
| ZNRF3 | zinc and ring finger 3 |
| ZSCAN29 | zinc finger and SCAN domain containing 29 |
|  |  |
|  |  |
