# Supplementary material for: Identification of significant genes and therapeutic agents for breast cancer by integrated genomics
Source: Bioengineered. 2021 Jun 21;12(1):2140–54. doi: 10.1080/21655979.2021.1931642 (PMC8806825; doi:10.1080/21655979.2021.1931642)
Supplement: Supplemental Material [file KBIE_A_1931642_SM7001.zip › supplementary2.docx]

**Top 5% Under-expressed genes in breast cancer**

| Gene symbol | Gene name |
| --- | --- |
| AADAC | arylacetamide deacetylase (esterase) |
| AADACL2 | arylacetamide deacetylase-like 2 |
| AASS | aminoadipate-semialdehyde synthase |
| ABCA6 | ATP-binding cassette, sub-family A (ABC1), member 6 |
| ABCA9 | ATP-binding cassette, sub-family A (ABC1), member 9 |
| ABCD2 | ATP-binding cassette, sub-family D (ALD), member 2 |
| ABHD14B | abhydrolase domain containing 14B |
| ACADL | acyl-CoA dehydrogenase, long chain |
| ACOT11 | acyl-CoA thioesterase 11 |
| ACOT2 | acyl-CoA thioesterase 2 |
| ACSS2 | acyl-CoA synthetase short-chain family member 2 |
| ACSS3 | acyl-CoA synthetase short-chain family member 3 |
| ACTBL2 | actin, beta-like 2 |
| ACVR1C | activin A receptor, type IC |
| ADAD2 | adenosine deaminase domain containing 2 |
| ADAMTS3 | ADAM metallopeptidase with thrombospondin type 1 motif, 3 |
| ADAMTSL3 | ADAMTS-like 3 |
| ADHFE1 | alcohol dehydrogenase, iron containing, 1 |
| ADRA1B | adrenergic, alpha-1B-, receptor |
| ADRA2B | adrenergic, alpha-2B-, receptor |
| AGAP4 | ArfGAP with GTPase domain, ankyrin repeat and PH domain 4 |
| AGAP5 | ArfGAP with GTPase domain, ankyrin repeat and PH domain 5 |
| AGAP9 | ArfGAP with GTPase domain, ankyrin repeat and PH domain 9 |
| AGBL4 | ATP/GTP binding protein-like 4 |
| AHSA2 | AHA1, activator of heat shock 90kDa protein ATPase homolog 2 (yeast) |
| AHSG | alpha-2-HS-glycoprotein |
| AK5 | adenylate kinase 5 |
| AKAP6 | A kinase (PRKA) anchor protein 6 |
| AKR1B15 | aldo-keto reductase family 1, member B15 |
| ALB | albumin |
| ALDH1A1 | aldehyde dehydrogenase 1 family, member A1 |
| ALDH1L1 | aldehyde dehydrogenase 1 family, member L1 |
| ALDH2 | aldehyde dehydrogenase 2 family (mitochondrial) |
| ALDH7A1 | aldehyde dehydrogenase 7 family, member A1 |
| ALK | anaplastic lymphoma receptor tyrosine kinase |
| ALOX12P2 | arachidonate 12-lipoxygenase pseudogene 2 |
| ALPK3 | alpha-kinase 3 |
| ALPL | alkaline phosphatase, liver/bone/kidney |
| ALPP | alkaline phosphatase, placental (Regan isozyme) |
| ALS2CR4 | amyotrophic lateral sclerosis 2 (juvenile) chromosome region, candidate 4 |
| AMOTL2 | angiomotin like 2 |
| AMPD1 | adenosine monophosphate deaminase 1 |
| AMT | aminomethyltransferase |
| ANAPC10 | anaphase promoting complex subunit 10 |
| ANGPT1 | angiopoietin 1 |
| ANGPTL1 | angiopoietin-like 1 |
| ANGPTL5 | angiopoietin-like 5 |
| ANGPTL7 | angiopoietin-like 7 |
| ANK2 | ankyrin 2, neuronal |
| ANKK1 | ankyrin repeat and kinase domain containing 1 |
| ANKRD27 | ankyrin repeat domain 27 (VPS9 domain) |
| ANKRD45 | ankyrin repeat domain 45 |
| ANKRD49 | ankyrin repeat domain 49 |
| ANKS6 | ankyrin repeat and sterile alpha motif domain containing 6 |
| ANO3 | anoctamin 3 |
| ANO4 | anoctamin 4 |
| ANO6 | anoctamin 6 |
| ANXA5 | annexin A5 |
| AOC2 | amine oxidase, copper containing 2 (retina-specific) |
| APBA1 | amyloid beta (A4) precursor protein-binding, family A, member 1 |
| APBB1 | amyloid beta (A4) precursor protein-binding, family B, member 1 (Fe65) |
| APIP | APAF1 interacting protein |
| APLP2 | amyloid beta (A4) precursor-like protein 2 |
| AQPEP | laeverin |
| ARHGAP19 | Rho GTPase activating protein 19 |
| ARHGEF12 | Rho guanine nucleotide exchange factor (GEF) 12 |
| ARHGEF4 | Rho guanine nucleotide exchange factor (GEF) 4 |
| ARID4A | AT rich interactive domain 4A (RBP1-like) |
| ARID5B | AT rich interactive domain 5B (MRF1-like) |
| ART5 | ADP-ribosyltransferase 5 |
| ASAH1 | N-acylsphingosine amidohydrolase (acid ceramidase) 1 |
| ASAM | adipocyte-specific adhesion molecule |
| ATF3 | activating transcription factor 3 |
| ATG4C | ATG4 autophagy related 4 homolog C (S. cerevisiae) |
| ATP11B | ATPase, class VI, type 11B |
| ATP13A4 | ATPase type 13A4 |
| ATP13A5 | ATPase type 13A5 |
| ATP4A | ATPase, H+/K+ exchanging, alpha polypeptide |
| ATP8A1 | ATPase, aminophospholipid transporter (APLT), class I, type 8A, member 1 |
| B3GNT1 | UDP-GlcNAc:betaGal beta-1,3-N-acetylglucosaminyltransferase 1 |
| BAI3 | brain-specific angiogenesis inhibitor 3 |
| BAT1 | HLA-B associated transcript 1 |
| BCHE | butyrylcholinesterase |
| BIN1 | bridging integrator 1 |
| BMPER | BMP binding endothelial regulator |
| BMS1P5 | BMS1 pseudogene 5 |
| BRMS1L | breast cancer metastasis-suppressor 1-like |
| BTN3A3 | butyrophilin, subfamily 3, member A3 |
| C10orf11 | chromosome 10 open reading frame 11 |
| C10orf54 | chromosome 10 open reading frame 54 |
| C10orf62 | chromosome 10 open reading frame 62 |
| C10orf90 | chromosome 10 open reading frame 90 |
| C11orf61 | chromosome 11 open reading frame 61 |
| C12orf39 | chromosome 12 open reading frame 39 |
| C12orf62 | chromosome 12 open reading frame 62 |
| C14orf23 | chromosome 14 open reading frame 23 |
| C14orf28 | chromosome 14 open reading frame 28 |
| C15orf51 | dynamin 1 pseudogene |
| C17orf49 | chromosome 17 open reading frame 49 |
| C17orf88 | chromosome 17 open reading frame 88 |
| C1orf123 | chromosome 1 open reading frame 123 |
| C1orf69 | chromosome 1 open reading frame 69 |
| C1QL4 | complement component 1, q subcomponent-like 4 |
| C1QTNF9 | C1q and tumor necrosis factor related protein 9 |
| C1QTNF9B | C1q and tumor necrosis factor related protein 9B |
| C20orf106 | chromosome 20 open reading frame 106 |
| C21orf29 | chromosome 21 open reading frame 29 |
| C2CD2 | C2 calcium-dependent domain containing 2 |
| C2orf56 | chromosome 2 open reading frame 56 |
| C2orf88 | chromosome 2 open reading frame 88 |
| C3orf58 | chromosome 3 open reading frame 58 |
| C3orf64 | chromosome 3 open reading frame 64 |
| C4BPB | complement component 4 binding protein, beta |
| C4orf12 | chromosome 4 open reading frame 12 |
| C4orf31 | chromosome 4 open reading frame 31 |
| C4orf33 | chromosome 4 open reading frame 33 |
| C5orf23 | chromosome 5 open reading frame 23 |
| C6orf10 | chromosome 6 open reading frame 10 |
| C6orf15 | chromosome 6 open reading frame 15 |
| C6orf58 | chromosome 6 open reading frame 58 |
| C6orf72 | chromosome 6 open reading frame 72 |
| C7orf34 | chromosome 7 open reading frame 34 |
| C7orf41 | chromosome 7 open reading frame 41 |
| C7orf45 | chromosome 7 open reading frame 45 |
| C8orf47 | chromosome 8 open reading frame 47 |
| C9orf21 | chromosome 9 open reading frame 21 |
| C9orf47 | chromosome 9 open reading frame 47 |
| C9orf6 | chromosome 9 open reading frame 6 |
| C9orf85 | chromosome 9 open reading frame 85 |
| CA4 | carbonic anhydrase IV |
| CABP1 | calcium binding protein 1 |
| CACNB1 | calcium channel, voltage-dependent, beta 1 subunit |
| CADM3 | cell adhesion molecule 3 |
| CAPN11 | calpain 11 |
| CAPRIN1 | cell cycle associated protein 1 |
| CARD8 | caspase recruitment domain family, member 8 |
| CASKIN2 | CASK interacting protein 2 |
| CASP10 | caspase 10, apoptosis-related cysteine peptidase |
| CAT | catalase |
| CBLN4 | cerebellin 4 precursor |
| CC2D1A | coiled-coil and C2 domain containing 1A |
| CCBL2 | cysteine conjugate-beta lyase 2 |
| CCDC36 | coiled-coil domain containing 36 |
| CCDC50 | coiled-coil domain containing 50 |
| CCDC55 | coiled-coil domain containing 55 |
| CCDC59 | coiled-coil domain containing 59 |
| CCDC66 | coiled-coil domain containing 66 |
| CCDC69 | coiled-coil domain containing 69 |
| CCDC76 | coiled-coil domain containing 76 |
| CCDC92 | coiled-coil domain containing 92 |
| CCL23 | chemokine (C-C motif) ligand 23 |
| CCNL1 | cyclin L1 |
| CCT4 | chaperonin containing TCP1, subunit 4 (delta) |
| CD160 | CD160 molecule |
| CD164 | CD164 molecule, sialomucin |
| CD300LG | CD300 molecule-like family member g |
| CD40 | CD40 molecule, TNF receptor superfamily member 5 |
| CD55 | CD55 molecule, decay accelerating factor for complement (Cromer blood group) |
| CDKN2C | cyclin-dependent kinase inhibitor 2C (p18, inhibits CDK4) |
| CEBPE | CCAAT/enhancer binding protein (C/EBP), epsilon |
| CES1 | carboxylesterase 1 (monocyte/macrophage serine esterase 1) |
| CES3 | carboxylesterase 3 |
| CFL2 | cofilin 2 (muscle) |
| CFLAR | CASP8 and FADD-like apoptosis regulator |
| CFTR | cystic fibrosis transmembrane conductance regulator (ATP-binding cassette sub-family C, member 7) |
| CHD9 | chromodomain helicase DNA binding protein 9 |
| CHODL | chondrolectin |
| CHP2 | calcineurin B homologous protein 2 |
| CHST2 | carbohydrate (N-acetylglucosamine-6-O) sulfotransferase 2 |
| CHST3 | carbohydrate (chondroitin 6) sulfotransferase 3 |
| CHST7 | carbohydrate (N-acetylglucosamine 6-O) sulfotransferase 7 |
| CHST9 | carbohydrate (N-acetylgalactosamine 4-0) sulfotransferase 9 |
| CIDEC | cell death-inducing DFFA-like effector c |
| CIDECP | cell death-inducing DFFA-like effector c pseudogene |
| CKMT2 | creatine kinase, mitochondrial 2 (sarcomeric) |
| CLCA2 | chloride channel accessory 2 |
| CLCA4 | chloride channel accessory 4 |
| CLCN4 | chloride channel 4 |
| CLDN10 | claudin 10 |
| CLDN11 | claudin 11 |
| CLEC2D | C-type lectin domain family 2, member D |
| CLIC4 | chloride intracellular channel 4 |
| CLIP3 | CAP-GLY domain containing linker protein 3 |
| CLK1 | CDC-like kinase 1 |
| CLK4 | CDC-like kinase 4 |
| CLU | clusterin |
| CMTM5 | CKLF-like MARVEL transmembrane domain containing 5 |
| CMYA5 | cardiomyopathy associated 5 |
| CNBP | CCHC-type zinc finger, nucleic acid binding protein |
| CNGA1 | cyclic nucleotide gated channel alpha 1 |
| CNGA2 | cyclic nucleotide gated channel alpha 2 |
| CNR1 | cannabinoid receptor 1 (brain) |
| CNTN1 | contactin 1 |
| CNTN3 | contactin 3 (plasmacytoma associated) |
| CNTN6 | contactin 6 |
| COBLL1 | COBL-like 1 |
| COL25A1 | collagen, type XXV, alpha 1 |
| COLEC12 | collectin sub-family member 12 |
| COQ3 | coenzyme Q3 homolog, methyltransferase (S. cerevisiae) |
| COQ9 | coenzyme Q9 homolog (S. cerevisiae) |
| COX10 | COX10 homolog, cytochrome c oxidase assembly protein, heme A: farnesyltransferase (yeast) |
| COX4I1 | cytochrome c oxidase subunit IV isoform 1 |
| CPA1 | carboxypeptidase A1 (pancreatic) |
| CPE | carboxypeptidase E |
| CPN2 | carboxypeptidase N, polypeptide 2 |
| CPNE8 | copine VIII |
| CREB3L3 | cAMP responsive element binding protein 3-like 3 |
| CRHBP | corticotropin releasing hormone binding protein |
| CRY2 | cryptochrome 2 (photolyase-like) |
| CSDE1 | cold shock domain containing E1, RNA-binding |
| CSF2RA | colony stimulating factor 2 receptor, alpha, low-affinity (granulocyte-macrophage) |
| CSGALNACT1 | chondroitin sulfate N-acetylgalactosaminyltransferase 1 |
| CSN1S1 | casein alpha s1 |
| CSN3 | casein kappa |
| CSRNP1 | cysteine-serine-rich nuclear protein 1 |
| CTAG1A | cancer/testis antigen 1A |
| CTAG1B | cancer/testis antigen 1B |
| CTDSPL | CTD (carboxy-terminal domain, RNA polymerase II, polypeptide A) small phosphatase-like |
| CTNNBL1 | catenin, beta like 1 |
| CTSW | cathepsin W |
| CTTNBP2NL | CTTNBP2 N-terminal like |
| CWF19L1 | CWF19-like 1, cell cycle control (S. pombe) |
| CX3CL1 | chemokine (C-X3-C motif) ligand 1 |
| CXCR2 | chemokine (C-X-C motif) receptor 2 |
| CYB5A | cytochrome b5 type A (microsomal) |
| CYBASC3 | cytochrome b, ascorbate dependent 3 |
| CYP11A1 | cytochrome P450, family 11, subfamily A, polypeptide 1 |
| CYP26B1 | cytochrome P450, family 26, subfamily B, polypeptide 1 |
| CYP39A1 | cytochrome P450, family 39, subfamily A, polypeptide 1 |
| CYP4A11 | cytochrome P450, family 4, subfamily A, polypeptide 11 |
| CYP4F11 | cytochrome P450, family 4, subfamily F, polypeptide 11 |
| CYP4F2 | cytochrome P450, family 4, subfamily F, polypeptide 2 |
| CYR61 | cysteine-rich, angiogenic inducer, 61 |
| DARS | aspartyl-tRNA synthetase |
| DCAF5 | DDB1 and CUL4 associated factor 5 |
| DDX1 | DEAD (Asp-Glu-Ala-Asp) box polypeptide 1 |
| DDX18 | DEAD (Asp-Glu-Ala-Asp) box polypeptide 18 |
| DDX19B | DEAD (Asp-Glu-Ala-As) box polypeptide 19B |
| DENND5A | DENN/MADD domain containing 5A |
| DHRS11 | dehydrogenase/reductase (SDR family) member 11 |
| DHRS3 | dehydrogenase/reductase (SDR family) member 3 |
| DIXDC1 | DIX domain containing 1 |
| DKK3 | dickkopf homolog 3 (Xenopus laevis) |
| DLD | dihydrolipoamide dehydrogenase |
| DLK1 | delta-like 1 homolog (Drosophila) |
| DLK2 | delta-like 2 homolog (Drosophila) |
| DLX3 | distal-less homeobox 3 |
| DMXL1 | Dmx-like 1 |
| DNAJB3 | DnaJ (Hsp40) homolog, subfamily B, member 3 |
| DNHD1 | dynein heavy chain domain 1 |
| DOCK11 | dedicator of cytokinesis 11 |
| DPH5 | DPH5 homolog (S. cerevisiae) |
| DPP4 | dipeptidyl-peptidase 4 |
| DPP6 | dipeptidyl-peptidase 6 |
| DRAM2 | DNA-damage regulated autophagy modulator 2 |
| DSTN | destrin (actin depolymerizing factor) |
| DUS3L | dihydrouridine synthase 3-like (S. cerevisiae) |
| DUSP1 | dual specificity phosphatase 1 |
| DYNC1LI2 | dynein, cytoplasmic 1, light intermediate chain 2 |
| EAPP | E2F-associated phosphoprotein |
| ECE2 | endothelin converting enzyme 2 |
| EDAR | ectodysplasin A receptor |
| EEF1A1 | eukaryotic translation elongation factor 1 alpha 1 |
| EEF1B2 | eukaryotic translation elongation factor 1 beta 2 |
| EEF1G | eukaryotic translation elongation factor 1 gamma |
| EGFL7 | EGF-like-domain, multiple 7 |
| EGFR | epidermal growth factor receptor (erythroblastic leukemia viral (v-erb-b) oncogene homolog, avian) |
| EHD2 | EH-domain containing 2 |
| EIF1 | eukaryotic translation initiation factor 1 |
| EIF3F | eukaryotic translation initiation factor 3, subunit F |
| EIF3K | eukaryotic translation initiation factor 3, subunit K |
| EIF3L | eukaryotic translation initiation factor 3, subunit L |
| EIF5A2 | eukaryotic translation initiation factor 5A2 |
| ELAC1 | elaC homolog 1 (E. coli) |
| ELOVL7 | ELOVL family member 7, elongation of long chain fatty acids (yeast) |
| EMP1 | epithelial membrane protein 1 |
| ENC1 | ectodermal-neural cortex 1 (with BTB-like domain) |
| ENDOU | endonuclease, polyU-specific |
| ENOSF1 | enolase superfamily member 1 |
| ENPP6 | ectonucleotide pyrophosphatase/phosphodiesterase 6 |
| EPAS1 | endothelial PAS domain protein 1 |
| EPB41L4A | erythrocyte membrane protein band 4.1 like 4A |
| EPB41L4B | erythrocyte membrane protein band 4.1 like 4B |
| EPC1 | enhancer of polycomb homolog 1 (Drosophila) |
| EPHB1 | EPH receptor B1 |
| EPM2A | epilepsy, progressive myoclonus type 2A, Lafora disease (laforin) |
| ERCC8 | excision repair cross-complementing rodent repair deficiency, complementation group 8 |
| ERG | v-ets erythroblastosis virus E26 oncogene homolog (avian) |
| ERP29 | endoplasmic reticulum protein 29 |
| ESD | esterase D |
| ETFA | electron-transfer-flavoprotein, alpha polypeptide |
| ETS2 | v-ets erythroblastosis virus E26 oncogene homolog 2 (avian) |
| ETV3L | ets variant 3-like |
| ETV6 | ets variant 6 |
| EXOC6B | exocyst complex component 6B |
| EXOC7 | exocyst complex component 7 |
| EXOSC6 | exosome component 6 |
| EXOSC8 | exosome component 8 |
| EXT2 | exostosin 2 |
| EXTL2 | exostoses (multiple)-like 2 |
| EZH1 | enhancer of zeste homolog 1 (Drosophila) |
| F10 | coagulation factor X |
| FABP7 | fatty acid binding protein 7, brain |
| FADS3 | fatty acid desaturase 3 |
| FAHD2A | fumarylacetoacetate hydrolase domain containing 2A |
| FAHD2B | fumarylacetoacetate hydrolase domain containing 2B |
| FAM107A | family with sequence similarity 107, member A |
| FAM108A1 | family with sequence similarity 108, member A1 |
| FAM10A4 | ST13-like tumor suppressor |
| FAM10A5 | family with sequence similarity 10, member A5 pseudogene |
| FAM134A | family with sequence similarity 134, member A |
| FAM13A | family with sequence similarity 13, member A |
| FAM13AOS | FAM13A opposite strand (non-protein coding) |
| FAM149A | family with sequence similarity 149, member A |
| FAM150B | family with sequence similarity 150, member B |
| FAM168A | family with sequence similarity 168, member A |
| FAM192A | family with sequence similarity 192, member A |
| FAM43A | family with sequence similarity 43, member A |
| FAM5C | family with sequence similarity 5, member C |
| FAM71A | family with sequence similarity 71, member A |
| FAM82A1 | family with sequence similarity 82, member A1 |
| FAM82B | family with sequence similarity 82, member B |
| FAM8A1 | family with sequence similarity 8, member A1 |
| FARSB | phenylalanyl-tRNA synthetase, beta subunit |
| FBLN1 | fibulin 1 |
| FBXO25 | F-box protein 25 |
| FBXW7 | F-box and WD repeat domain containing 7 |
| FDX1 | ferredoxin 1 |
| FER1L5 | fer-1-like 5 (C. elegans) |
| FERMT2 | fermitin family homolog 2 (Drosophila) |
| FEZF2 | FEZ family zinc finger 2 |
| FGF2 | fibroblast growth factor 2 (basic) |
| FGF7 | fibroblast growth factor 7 (keratinocyte growth factor) |
| FGF9 | fibroblast growth factor 9 (glia-activating factor) |
| FGFBP1 | fibroblast growth factor binding protein 1 |
| FGG | fibrinogen gamma chain |
| FGR | Gardner-Rasheed feline sarcoma viral (v-fgr) oncogene homolog |
| FHL5 | four and a half LIM domains 5 |
| FLII | flightless I homolog (Drosophila) |
| FLJ10038 | hypothetical protein FLJ10038 |
| FLJ35024 | hypothetical LOC401491 |
| FLJ35946 | hypothetical protein FLJ35946 |
| FLJ36031 | hypothetical protein FLJ36031 |
| FLJ38723 | hypothetical FLJ38723 |
| FLJ42875 | hypothetical LOC440556 |
| FNDC3B | fibronectin type III domain containing 3B |
| FOLR1 | folate receptor 1 (adult) |
| FOLR2 | folate receptor 2 (fetal) |
| FOXC1 | forkhead box C1 |
| FOXE1 | forkhead box E1 (thyroid transcription factor 2) |
| FOXN3 | forkhead box N3 |
| FRMPD1 | FERM and PDZ domain containing 1 |
| FTMT | ferritin mitochondrial |
| FUT9 | fucosyltransferase 9 (alpha (1,3) fucosyltransferase) |
| FXYD1 | FXYD domain containing ion transport regulator 1 |
| FXYD2 | FXYD domain containing ion transport regulator 2 |
| GABRR3 | gamma-aminobutyric acid (GABA) receptor, rho 3 |
| GADD45B | growth arrest and DNA-damage-inducible, beta |
| GALT | galactose-1-phosphate uridylyltransferase |
| GDPD5 | glycerophosphodiester phosphodiesterase domain containing 5 |
| GFRA2 | GDNF family receptor alpha 2 |
| GIPC2 | GIPC PDZ domain containing family, member 2 |
| GJA9 | gap junction protein, alpha 9, 59kDa |
| GLP2R | glucagon-like peptide 2 receptor |
| GLRA3 | glycine receptor, alpha 3 |
| GLYAT | glycine-N-acyltransferase |
| GLYATL2 | glycine-N-acyltransferase-like 2 |
| GNE | glucosamine (UDP-N-acetyl)-2-epimerase/N-acetylmannosamine kinase |
| GNG7 | guanine nucleotide binding protein (G protein), gamma 7 |
| GOLGA4 | golgin A4 |
| GOLGA6L10 | golgin A6 family-like 10 |
| GP1BA | glycoprotein Ib (platelet), alpha polypeptide |
| GPAM | glycerol-3-phosphate acyltransferase, mitochondrial |
| GPC3 | glypican 3 |
| GPCPD1 | glycerophosphocholine phosphodiesterase GDE1 homolog (S. cerevisiae) |
| GPD1 | glycerol-3-phosphate dehydrogenase 1 (soluble) |
| GPLD1 | glycosylphosphatidylinositol specific phospholipase D1 |
| GPM6A | glycoprotein M6A |
| GPR116 | G protein-coupled receptor 116 |
| GPR146 | G protein-coupled receptor 146 |
| GPR180 | G protein-coupled receptor 180 |
| GPR88 | G protein-coupled receptor 88 |
| GPRC5B | G protein-coupled receptor, family C, group 5, member B |
| GRHL1 | grainyhead-like 1 (Drosophila) |
| GRIA2 | glutamate receptor, ionotropic, AMPA 2 |
| GRIK1 | glutamate receptor, ionotropic, kainate 1 |
| GRK5 | G protein-coupled receptor kinase 5 |
| GSDMA | gasdermin A |
| GSN | gelsolin |
| GSTA2 | glutathione S-transferase alpha 2 |
| GSTA5 | glutathione S-transferase alpha 5 |
| GSTM2 | glutathione S-transferase mu 2 (muscle) |
| GSTM5 | glutathione S-transferase mu 5 |
| GUCA1B | guanylate cyclase activator 1B (retina) |
| GXYLT1 | glucoside xylosyltransferase 1 |
| HADH | hydroxyacyl-CoA dehydrogenase |
| HADHA | hydroxyacyl-CoA dehydrogenase/3-ketoacyl-CoA thiolase/enoyl-CoA hydratase (trifunctional protein), alpha subunit |
| HBD | hemoglobin, delta |
| HBG1 | hemoglobin, gamma A |
| HBG2 | hemoglobin, gamma G |
| HCFC2 | host cell factor C2 |
| HEPACAM | hepatocyte cell adhesion molecule |
| HEPN1 | HEPACAM opposite strand 1 |
| HERPUD1 | homocysteine-inducible, endoplasmic reticulum stress-inducible, ubiquitin-like domain member 1 |
| HESX1 | HESX homeobox 1 |
| HIBADH | 3-hydroxyisobutyrate dehydrogenase |
| HIF3A | hypoxia inducible factor 3, alpha subunit |
| HLA-DPA1 | major histocompatibility complex, class II, DP alpha 1 |
| HNRNPA1L2 | heterogeneous nuclear ribonucleoprotein A1-like 2 |
| HNRNPH1 | heterogeneous nuclear ribonucleoprotein H1 (H) |
| HOXA2 | homeobox A2 |
| HOXA3 | homeobox A3 |
| HOXA9 | homeobox A9 |
| HOXD10 | homeobox D10 |
| HPD | 4-hydroxyphenylpyruvate dioxygenase |
| HRCT1 | histidine rich carboxyl terminus 1 |
| HS3ST4 | heparan sulfate (glucosamine) 3-O-sulfotransferase 4 |
| HSPB7 | heat shock 27kDa protein family, member 7 (cardiovascular) |
| HSPC072 | hypothetical LOC29075 |
| HTRA2 | HtrA serine peptidase 2 |
| IFFO2 | intermediate filament family orphan 2 |
| IFRD1 | interferon-related developmental regulator 1 |
| IGF1 | insulin-like growth factor 1 (somatomedin C) |
| IGFBP1 | insulin-like growth factor binding protein 1 |
| IGFBP6 | insulin-like growth factor binding protein 6 |
| IGHA1 | immunoglobulin heavy constant alpha 1 |
| IGJ | immunoglobulin J polypeptide, linker protein for immunoglobulin alpha and mu polypeptides |
| IGKC | immunoglobulin kappa constant |
| IGKV1-5 | immunoglobulin kappa variable 1-5 |
| IGKV2-24 | immunoglobulin kappa variable 2-24 |
| IGL@ | immunoglobulin lambda locus |
| IGLL1 | immunoglobulin lambda-like polypeptide 1 |
| IGLL3 | immunoglobulin lambda-like polypeptide 3 |
| IGLV2-14 | immunoglobulin lambda variable 2-14 |
| IGLV3-25 | immunoglobulin lambda variable 3-25 |
| IGLV6-57 | immunoglobulin lambda variable 6-57 |
| IL1F10 | interleukin 1 family, member 10 (theta) |
| IL1RL1 | interleukin 1 receptor-like 1 |
| IL2 | interleukin 2 |
| IL20RB | interleukin 20 receptor beta |
| IL22RA2 | interleukin 22 receptor, alpha 2 |
| IL3RA | interleukin 3 receptor, alpha (low affinity) |
| IL6 | interleukin 6 (interferon, beta 2) |
| ILDR1 | immunoglobulin-like domain containing receptor 1 |
| IPO5 | importin 5 |
| IQCF2 | IQ motif containing F2 |
| IRX6 | iroquois homeobox 6 |
| ISL1 | ISL LIM homeobox 1 |
| ISLR2 | immunoglobulin superfamily containing leucine-rich repeat 2 |
| ITIH2 | inter-alpha (globulin) inhibitor H2 |
| ITIH3 | inter-alpha (globulin) inhibitor H3 |
| ITIH5 | inter-alpha (globulin) inhibitor H5 |
| ITM2C | integral membrane protein 2C |
| JMJD1C | jumonji domain containing 1C |
| JUNB | jun B proto-oncogene |
| KANK1 | KN motif and ankyrin repeat domains 1 |
| KANK2 | KN motif and ankyrin repeat domains 2 |
| KAT2B | K(lysine) acetyltransferase 2B |
| KATNB1 | katanin p80 (WD repeat containing) subunit B 1 |
| KCNA1 | potassium voltage-gated channel, shaker-related subfamily, member 1 (episodic ataxia with myokymia) |
| KCNA2 | potassium voltage-gated channel, shaker-related subfamily, member 2 |
| KCNAB1 | potassium voltage-gated channel, shaker-related subfamily, beta member 1 |
| KCNC1 | potassium voltage-gated channel, Shaw-related subfamily, member 1 |
| KCNE1L | KCNE1-like |
| KCNH6 | potassium voltage-gated channel, subfamily H (eag-related), member 6 |
| KCNH8 | potassium voltage-gated channel, subfamily H (eag-related), member 8 |
| KCNJ1 | potassium inwardly-rectifying channel, subfamily J, member 1 |
| KCNJ16 | potassium inwardly-rectifying channel, subfamily J, member 16 |
| KCNK3 | potassium channel, subfamily K, member 3 |
| KCTD14 | potassium channel tetramerisation domain containing 14 |
| KGFLP1 | keratinocyte growth factor-like protein 1 |
| KGFLP2 | keratinocyte growth factor-like protein 2 |
| KIAA0664 | KIAA0664 |
| KIAA1161 | KIAA1161 |
| KIAA1984 | KIAA1984 |
| KIAA2022 | KIAA2022 |
| KIT | v-kit Hardy-Zuckerman 4 feline sarcoma viral oncogene homolog |
| KL | klotho |
| KLB | klotho beta |
| KLF15 | Kruppel-like factor 15 |
| KLF6 | Kruppel-like factor 6 |
| KLHL3 | kelch-like 3 (Drosophila) |
| KLHL34 | kelch-like 34 (Drosophila) |
| KLHL4 | kelch-like 4 (Drosophila) |
| KLK5 | kallikrein-related peptidase 5 |
| KLK7 | kallikrein-related peptidase 7 |
| KLKB1 | kallikrein B, plasma (Fletcher factor) 1 |
| KLRF1 | killer cell lectin-like receptor subfamily F, member 1 |
| KRT16P3 | keratin 16 pseudogene 3 |
| KY | kyphoscoliosis peptidase |
| L3MBTL4 | l(3)mbt-like 4 (Drosophila) |
| LARP7 | La ribonucleoprotein domain family, member 7 |
| LATS1 | LATS, large tumor suppressor, homolog 1 (Drosophila) |
| LDB3 | LIM domain binding 3 |
| LEFTY2 | left-right determination factor 2 |
| LENEP | lens epithelial protein |
| LEPR | leptin receptor |
| LGALS12 | lectin, galactoside-binding, soluble, 12 |
| LGALS4 | lectin, galactoside-binding, soluble, 4 |
| LGI4 | leucine-rich repeat LGI family, member 4 |
| LILRA5 | leukocyte immunoglobulin-like receptor, subfamily A (with TM domain), member 5 |
| LMBRD2 | LMBR1 domain containing 2 |
| LOC100128175 | similar to PRO2591 |
| LOC100128893 | hypothetical protein LOC100128893 |
| LOC100129397 | hypothetical protein LOC100129397 |
| LOC100129794 | similar to hCG1804255 |
| LOC100130100 | similar to hCG26659 |
| LOC100130107 | hypothetical LOC100130107 |
| LOC100131699 | hypothetical LOC100131699 |
| LOC100131825 | hypothetical protein LOC100131825 |
| LOC100131943 | RVLA1944 |
| LOC100132540 | similar to LOC339047 protein |
| LOC100132705 | similar to immunoglobulin superfamily, member 3 |
| LOC100132771 | hypothetical LOC100132771 |
| LOC100132941 | similar to Ig heavy chain |
| LOC100133177 | hypothetical LOC100133177 |
| LOC100134397 | similar to hCG1812074 |
| LOC100190939 | hypothetical LOC100190939 |
| LOC100192379 | hypothetical LOC100192379 |
| LOC100288394 | hypothetical protein LOC100288394 |
| LOC120364 | similar to heterogeneous nuclear ribonucleoprotein A1-like |
| LOC134505 | similar to hCG39609 |
| LOC151438 | hypothetical protein LOC151438 |
| LOC152217 | hypothetical LOC152217 |
| LOC158257 | hypothetical protein LOC158257 |
| LOC253039 | hypothetical LOC253039 |
| LOC280665 | anti-CNG alpha 1 cation channel translation product-like |
| LOC283481 | hypothetical protein LOC283481 |
| LOC284112 | hypothetical protein LOC284112 |
| LOC284998 | hypothetical protein LOC284998 |
| LOC285831 | hypothetical protein LOC285831 |
| LOC349114 | hypothetical LOC349114 |
| LOC375196 | hypothetical protein LOC375196 |
| LOC389332 | hypothetical LOC389332 |
| LOC389705 | chromosome 4 open reading frame 27 pseudogene |
| LOC390282 | similar to hCG2040283 |
| LOC399491 | GPS, PLAT and transmembrane domain-containing protein |
| LOC440104 | hypothetical LOC440104 |
| LOC440434 | hypothetical protein FLJ11822 |
| LOC541472 | hypothetical LOC541472 |
| LOC55908 | hepatocellular carcinoma-associated gene TD26 |
| LOC572558 | hypothetical locus LOC572558 |
| LOC642131 | similar to hCG1812074 |
| LOC642132 | similar to roundabout 1 isoform b |
| LOC643310 | similar to heat shock 70kD protein binding protein |
| LOC643637 | similar to hCG1729961 |
| LOC644662 | similar to hCG2042541 |
| LOC646057 | similar to hCG2003024 |
| LOC647030 | eukaryotic translation elongation factor 1 beta 2-like |
| LOC647979 | hypothetical LOC647979 |
| LOC653097 | similar to hCG1988827 |
| LOC654780 | SFPQ |
| LOC729143 | similar to Myosin phosphatase Rho-interacting protein (Rho-interacting protein 3) (M-RIP) (RIP3) (p116Rip) |
| LOC729234 | fumarylacetoacetate hydrolase domain containing 2 pseudogene |
| LOC729342 | similar to nucleophosmin 1 |
| LOC729602 | NPIP-like protein ENSP00000283050 |
| LOC729992 | similar to heat shock 70kD protein binding protein |
| LOC730144 | similar to eukaryotic translation initiation factor 1 |
| LOC780529 | hypothetical LOC780529 |
| LOR | loricrin |
| LOXL4 | lysyl oxidase-like 4 |
| LPA | lipoprotein, Lp(a) |
| LRP12 | low density lipoprotein receptor-related protein 12 |
| LRP1B | low density lipoprotein receptor-related protein 1B |
| LRRC2 | leucine rich repeat containing 2 |
| LRRC9 | leucine rich repeat containing 9 |
| LRRFIP1 | leucine rich repeat (in FLII) interacting protein 1 |
| LRRN3 | leucine rich repeat neuronal 3 |
| LRRTM2 | leucine rich repeat transmembrane neuronal 2 |
| LSM14A | LSM14A, SCD6 homolog A (S. cerevisiae) |
| LY6D | lymphocyte antigen 6 complex, locus D |
| LY75 | lymphocyte antigen 75 |
| LY9 | lymphocyte antigen 9 |
| MAN2A2 | mannosidase, alpha, class 2A, member 2 |
| MAP1LC3C | microtubule-associated protein 1 light chain 3 gamma |
| MAP2 | microtubule-associated protein 2 |
| MAP3K3 | mitogen-activated protein kinase kinase kinase 3 |
| MAP3K5 | mitogen-activated protein kinase kinase kinase 5 |
| MAPK4 | mitogen-activated protein kinase 4 |
| MATN2 | matrilin 2 |
| MBTPS1 | membrane-bound transcription factor peptidase, site 1 |
| MCAM | melanoma cell adhesion molecule |
| MCCC1 | methylcrotonoyl-CoA carboxylase 1 (alpha) |
| MDFIC | MyoD family inhibitor domain containing |
| MED28 | mediator complex subunit 28 |
| MEG3 | maternally expressed 3 (non-protein coding) |
| METAP1 | methionyl aminopeptidase 1 |
| MGAT4C | mannosyl (alpha-1,3-)-glycoprotein beta-1,4-N-acetylglucosaminyltransferase, isozyme C (putative) |
| MITF | microphthalmia-associated transcription factor |
| MMAB | methylmalonic aciduria (cobalamin deficiency) cblB type |
| MMP19 | matrix metallopeptidase 19 |
| MMP28 | matrix metallopeptidase 28 |
| MMRN1 | multimerin 1 |
| MON2 | MON2 homolog (S. cerevisiae) |
| MORC3 | MORC family CW-type zinc finger 3 |
| MOSC1 | MOCO sulphurase C-terminal domain containing 1 |
| MPPED2 | metallophosphoesterase domain containing 2 |
| MRAP2 | melanocortin 2 receptor accessory protein 2 |
| MRPL39 | mitochondrial ribosomal protein L39 |
| MRPS31 | mitochondrial ribosomal protein S31 |
| MRPS5 | mitochondrial ribosomal protein S5 |
| MS4A6E | membrane-spanning 4-domains, subfamily A, member 6E |
| MSRB3 | methionine sulfoxide reductase B3 |
| MST1 | macrophage stimulating 1 (hepatocyte growth factor-like) |
| MST1P9 | macrophage stimulating 1 (hepatocyte growth factor-like) pseudogene 9 |
| MSTN | myostatin |
| MTERFD3 | MTERF domain containing 3 |
| MTUS1 | microtubule associated tumor suppressor 1 |
| MUC4 | mucin 4, cell surface associated |
| MUCL1 | mucin-like 1 |
| MYC | v-myc myelocytomatosis viral oncogene homolog (avian) |
| MYH10 | myosin, heavy chain 10, non-muscle |
| MYH7B | myosin, heavy chain 7B, cardiac muscle, beta |
| MYL6 | myosin, light chain 6, alkali, smooth muscle and non-muscle |
| MYOC | myocilin, trabecular meshwork inducible glucocorticoid response |
| MYOT | myotilin |
| NACA | nascent polypeptide-associated complex alpha subunit |
| NAMPT | nicotinamide phosphoribosyltransferase |
| NCAM1 | neural cell adhesion molecule 1 |
| NCOA1 | nuclear receptor coactivator 1 |
| NDRG2 | NDRG family member 2 |
| NEDD9 | neural precursor cell expressed, developmentally down-regulated 9 |
| NEUROG2 | neurogenin 2 |
| NFATC3 | nuclear factor of activated T-cells, cytoplasmic, calcineurin-dependent 3 |
| NFKBID | nuclear factor of kappa light polypeptide gene enhancer in B-cells inhibitor, delta |
| NGFR | nerve growth factor receptor |
| NIP7 | nuclear import 7 homolog (S. cerevisiae) |
| NKX2-1 | NK2 homeobox 1 |
| NKX2-8 | NK2 homeobox 8 |
| NMT2 | N-myristoyltransferase 2 |
| NOL4 | nucleolar protein 4 |
| NOV | nephroblastoma overexpressed gene |
| NPHP3 | nephronophthisis 3 (adolescent) |
| NPM1 | nucleophosmin (nucleolar phosphoprotein B23, numatrin) |
| NPR2 | natriuretic peptide receptor B/guanylate cyclase B (atrionatriuretic peptide receptor B) |
| NPR3 | natriuretic peptide receptor C/guanylate cyclase C (atrionatriuretic peptide receptor C) |
| NPY2R | neuropeptide Y receptor Y2 |
| NR1H3 | nuclear receptor subfamily 1, group H, member 3 |
| NR4A3 | nuclear receptor subfamily 4, group A, member 3 |
| NRXN1 | neurexin 1 |
| NUBPL | nucleotide binding protein-like |
| NUDT19 | nudix (nucleoside diphosphate linked moiety X)-type motif 19 |
| OBFC2A | oligonucleotide/oligosaccharide-binding fold containing 2A |
| OCM | oncomodulin |
| OCM2 | oncomodulin 2 |
| ODAM | odontogenic, ameloblast asssociated |
| OGN | osteoglycin |
| OLFM4 | olfactomedin 4 |
| OLIG1 | oligodendrocyte transcription factor 1 |
| OR4F17 | olfactory receptor, family 4, subfamily F, member 17 |
| OR4F4 | olfactory receptor, family 4, subfamily F, member 4 |
| OR5D18 | olfactory receptor, family 5, subfamily D, member 18 |
| OR5F1 | olfactory receptor, family 5, subfamily F, member 1 |
| OR6Y1 | olfactory receptor, family 6, subfamily Y, member 1 |
| OSBPL7 | oxysterol binding protein-like 7 |
| OXCT2 | 3-oxoacid CoA transferase 2 |
| OXTR | oxytocin receptor |
| P2RX1 | purinergic receptor P2X, ligand-gated ion channel, 1 |
| PABPC5 | poly(A) binding protein, cytoplasmic 5 |
| PAK7 | p21 protein (Cdc42/Rac)-activated kinase 7 |
| PARVA | parvin, alpha |
| PAX4 | paired box 4 |
| PC | pyruvate carboxylase |
| PCDH10 | protocadherin 10 |
| PCDH15 | protocadherin-related 15 |
| PCDH9 | protocadherin 9 |
| PCK1 | phosphoenolpyruvate carboxykinase 1 (soluble) |
| PCTP | phosphatidylcholine transfer protein |
| PCYOX1 | prenylcysteine oxidase 1 |
| PDE9A | phosphodiesterase 9A |
| PDK4 | pyruvate dehydrogenase kinase, isozyme 4 |
| PDLIM3 | PDZ and LIM domain 3 |
| PDXK | pyridoxal (pyridoxine, vitamin B6) kinase |
| PDZD2 | PDZ domain containing 2 |
| PEMT | phosphatidylethanolamine N-methyltransferase |
| PENK | proenkephalin |
| PER2 | period homolog 2 (Drosophila) |
| PFDN5 | prefoldin subunit 5 |
| PFKFB1 | 6-phosphofructo-2-kinase/fructose-2,6-biphosphatase 1 |
| PFKFB3 | 6-phosphofructo-2-kinase/fructose-2,6-biphosphatase 3 |
| PHC3 | polyhomeotic homolog 3 (Drosophila) |
| PHF17 | PHD finger protein 17 |
| PHIP | pleckstrin homology domain interacting protein |
| PI3 | peptidase inhibitor 3, skin-derived |
| PIGR | polymeric immunoglobulin receptor |
| PIK3R1 | phosphoinositide-3-kinase, regulatory subunit 1 (alpha) |
| PIK3R5 | phosphoinositide-3-kinase, regulatory subunit 5 |
| PINK1 | PTEN induced putative kinase 1 |
| PKD1L2 | polycystic kidney disease 1-like 2 |
| PKHD1L1 | polycystic kidney and hepatic disease 1 (autosomal recessive)-like 1 |
| PKN3 | protein kinase N3 |
| PLA2G3 | phospholipase A2, group III |
| PLA2G5 | phospholipase A2, group V |
| PLB1 | phospholipase B1 |
| PLCB1 | phospholipase C, beta 1 (phosphoinositide-specific) |
| PLD2 | phospholipase D2 |
| PLEKHG4 | pleckstrin homology domain containing, family G (with RhoGef domain) member 4 |
| PLEKHM1 | pleckstrin homology domain containing, family M (with RUN domain) member 1 |
| PLLP | plasma membrane proteolipid (plasmolipin) |
| PLSCR4 | phospholipid scramblase 4 |
| PLXNA4 | plexin A4 |
| PMS1 | PMS1 postmeiotic segregation increased 1 (S. cerevisiae) |
| PNMAL2 | PNMA-like 2 |
| PNPLA2 | patatin-like phospholipase domain containing 2 |
| PNRC1 | proline-rich nuclear receptor coactivator 1 |
| POLH | polymerase (DNA directed), eta |
| POLR1E | polymerase (RNA) I polypeptide E, 53kDa |
| PPA2 | pyrophosphatase (inorganic) 2 |
| PPARGC1B | peroxisome proliferator-activated receptor gamma, coactivator 1 beta |
| PPP1R15A | protein phosphatase 1, regulatory (inhibitor) subunit 15A |
| PPP2R1B | protein phosphatase 2, regulatory subunit A, beta |
| PRB1 | proline-rich protein BstNI subfamily 1 |
| PRB2 | proline-rich protein BstNI subfamily 2 |
| PRCP | prolylcarboxypeptidase (angiotensinase C) |
| PRDM1 | PR domain containing 1, with ZNF domain |
| PRDM10 | PR domain containing 10 |
| PRELP | proline/arginine-rich end leucine-rich repeat protein |
| PREPL | prolyl endopeptidase-like |
| PRG2 | proteoglycan 2, bone marrow (natural killer cell activator, eosinophil granule major basic protein) |
| PRH2 | proline-rich protein HaeIII subfamily 2 |
| PRMT10 | protein arginine methyltransferase 10 (putative) |
| PRNP | prion protein |
| PROL1 | proline rich, lacrimal 1 |
| PROS1 | protein S (alpha) |
| PRPF8 | PRP8 pre-mRNA processing factor 8 homolog (S. cerevisiae) |
| PRR4 | proline rich 4 (lacrimal) |
| PRRG3 | proline rich Gla (G-carboxyglutamic acid) 3 (transmembrane) |
| PRSS38 | protease, serine, 38 |
| PSG3 | pregnancy specific beta-1-glycoprotein 3 |
| PTCHD1 | patched domain containing 1 |
| PTGER4 | prostaglandin E receptor 4 (subtype EP4) |
| PTGES3 | prostaglandin E synthase 3 (cytosolic) |
| PTGS2 | prostaglandin-endoperoxide synthase 2 (prostaglandin G/H synthase and cyclooxygenase) |
| PTH2R | parathyroid hormone 2 receptor |
| PTRF | polymerase I and transcript release factor |
| PTX3 | pentraxin 3, long |
| PURG | purine-rich element binding protein G |
| PXN | paxillin |
| RABGAP1L | RAB GTPase activating protein 1-like |
| RANBP2 | RAN binding protein 2 |
| RAPGEF3 | Rap guanine nucleotide exchange factor (GEF) 3 |
| RASD2 | RASD family, member 2 |
| RASSF8 | Ras association (RalGDS/AF-6) domain family (N-terminal) member 8 |
| RBM15B | RNA binding motif protein 15B |
| RBP4 | retinol binding protein 4, plasma |
| RCAN1 | regulator of calcineurin 1 |
| RCN1 | reticulocalbin 1, EF-hand calcium binding domain |
| RECK | reversion-inducing-cysteine-rich protein with kazal motifs |
| RETSAT | retinol saturase (all-trans-retinol 13,14-reductase) |
| REV1 | REV1 homolog (S. cerevisiae) |
| REXO2 | REX2, RNA exonuclease 2 homolog (S. cerevisiae) |
| RFESD | Rieske (Fe-S) domain containing |
| RGL1 | ral guanine nucleotide dissociation stimulator-like 1 |
| RGNEF | 190 kDa guanine nucleotide exchange factor |
| RGPD8 | RANBP2-like and GRIP domain containing 8 |
| RGS2 | regulator of G-protein signaling 2, 24kDa |
| RGS7BP | regulator of G-protein signaling 7 binding protein |
| RICTOR | RPTOR independent companion of MTOR, complex 2 |
| RIN3 | Ras and Rab interactor 3 |
| RIPPLY2 | ripply2 homolog (zebrafish) |
| RNASE4 | ribonuclease, RNase A family, 4 |
| RNASE7 | ribonuclease, RNase A family, 7 |
| RNF125 | ring finger protein 125 |
| RNF130 | ring finger protein 130 |
| RNLS | renalase, FAD-dependent amine oxidase |
| ROBO1 | roundabout, axon guidance receptor, homolog 1 (Drosophila) |
| ROBO2 | roundabout, axon guidance receptor, homolog 2 (Drosophila) |
| ROPN1 | ropporin, rhophilin associated protein 1 |
| RPL18 | ribosomal protein L18 |
| RPL26 | ribosomal protein L26 |
| RPL26L1 | ribosomal protein L26-like 1 |
| RPL32 | ribosomal protein L32 |
| RPL35 | ribosomal protein L35 |
| RPL9 | ribosomal protein L9 |
| RPS13 | ribosomal protein S13 |
| RPS3 | ribosomal protein S3 |
| RPS6 | ribosomal protein S6 |
| RPS6KA3 | ribosomal protein S6 kinase, 90kDa, polypeptide 3 |
| RPS6P6 | ribosomal protein S6 pseudogene 6 |
| RRAS | related RAS viral (r-ras) oncogene homolog |
| RRN3P1 | RNA polymerase I transcription factor homolog (S. cerevisiae) pseudogene 1 |
| RSL24D1 | ribosomal L24 domain containing 1 |
| RUSC2 | RUN and SH3 domain containing 2 |
| RYR3 | ryanodine receptor 3 |
| S100A4 | S100 calcium binding protein A4 |
| S100B | S100 calcium binding protein B |
| S1PR3 | sphingosine-1-phosphate receptor 3 |
| SAA3P | serum amyloid A3 pseudogene |
| SAA4 | serum amyloid A4, constitutive |
| SARDH | sarcosine dehydrogenase |
| SBDS | Shwachman-Bodian-Diamond syndrome |
| SBDSP1 | Shwachman-Bodian-Diamond syndrome pseudogene 1 |
| SCAI | suppressor of cancer cell invasion |
| SCHIP1 | schwannomin interacting protein 1 |
| SCN2A | sodium channel, voltage-gated, type II, alpha subunit |
| SCN2B | sodium channel, voltage-gated, type II, beta |
| SCN4A | sodium channel, voltage-gated, type IV, alpha subunit |
| SCTR | secretin receptor |
| SDHAF2 | succinate dehydrogenase complex assembly factor 2 |
| SEC24A | SEC24 family, member A (S. cerevisiae) |
| SEC63 | SEC63 homolog (S. cerevisiae) |
| SEMA3G | sema domain, immunoglobulin domain (Ig), short basic domain, secreted, (semaphorin) 3G |
| SENP3 | SUMO1/sentrin/SMT3 specific peptidase 3 |
| SENP6 | SUMO1/sentrin specific peptidase 6 |
| SEPSECS | Sep (O-phosphoserine) tRNA:Sec (selenocysteine) tRNA synthase |
| 7-Sep | septin 7 |
| SERBP1 | SERPINE1 mRNA binding protein 1 |
| SERINC3 | serine incorporator 3 |
| SERPINB5 | serpin peptidase inhibitor, clade B (ovalbumin), member 5 |
| SETMAR | SET domain and mariner transposase fusion gene |
| SFRS11 | splicing factor, arginine/serine-rich 11 |
| SFRS13B | splicing factor, arginine/serine-rich 13B |
| SFRS16 | splicing factor, arginine/serine-rich 16 |
| SFRS2B | splicing factor, arginine/serine-rich 2B |
| SFRS5 | splicing factor, arginine/serine-rich 5 |
| SFTPB | surfactant protein B |
| SGCG | sarcoglycan, gamma (35kDa dystrophin-associated glycoprotein) |
| SGK2 | serum/glucocorticoid regulated kinase 2 |
| SH3D19 | SH3 domain containing 19 |
| SH3GLB1 | SH3-domain GRB2-like endophilin B1 |
| SH3KBP1 | SH3-domain kinase binding protein 1 |
| SIK1 | salt-inducible kinase 1 |
| SIX4 | SIX homeobox 4 |
| SIX5 | SIX homeobox 5 |
| SLC10A7 | solute carrier family 10 (sodium/bile acid cotransporter family), member 7 |
| SLC14A1 | solute carrier family 14 (urea transporter), member 1 (Kidd blood group) |
| SLC14A2 | solute carrier family 14 (urea transporter), member 2 |
| SLC15A4 | solute carrier family 15, member 4 |
| SLC16A8 | solute carrier family 16, member 8 (monocarboxylic acid transporter 3) |
| SLC25A1 | solute carrier family 25 (mitochondrial carrier; citrate transporter), member 1 |
| SLC25A32 | solute carrier family 25, member 32 |
| SLC25A42 | solute carrier family 25, member 42 |
| SLC26A4 | solute carrier family 26, member 4 |
| SLC2A12 | solute carrier family 2 (facilitated glucose transporter), member 12 |
| SLC2A3 | solute carrier family 2 (facilitated glucose transporter), member 3 |
| SLC35E4 | solute carrier family 35, member E4 |
| SLC38A4 | solute carrier family 38, member 4 |
| SLC3A1 | solute carrier family 3 (cystine, dibasic and neutral amino acid transporters, activator of cystine, dibasic and neutral amino acid transport), member 1 |
| SLC43A3 | solute carrier family 43, member 3 |
| SLC4A4 | solute carrier family 4, sodium bicarbonate cotransporter, member 4 |
| SLC6A14 | solute carrier family 6 (amino acid transporter), member 14 |
| SLC6A2 | solute carrier family 6 (neurotransmitter transporter, noradrenalin), member 2 |
| SLCO1A2 | solute carrier organic anion transporter family, member 1A2 |
| SMCR5 | Smith-Magenis syndrome chromosome region, candidate 5 (non-protein coding) |
| SMN1 | survival of motor neuron 1, telomeric |
| SMN2 | survival of motor neuron 2, centromeric |
| SMR3B | submaxillary gland androgen regulated protein 3B |
| SMYD4 | SET and MYND domain containing 4 |
| SNRNP48 | small nuclear ribonucleoprotein 48kDa (U11/U12) |
| SNRPN | small nuclear ribonucleoprotein polypeptide N |
| SNTB2 | syntrophin, beta 2 (dystrophin-associated protein A1, 59kDa, basic component 2) |
| SNX21 | sorting nexin family member 21 |
| SOCS3 | suppressor of cytokine signaling 3 |
| SOHLH2 | spermatogenesis and oogenesis specific basic helix-loop-helix 2 |
| SORBS1 | sorbin and SH3 domain containing 1 |
| SOX10 | SRY (sex determining region Y)-box 10 |
| SOX7 | SRY (sex determining region Y)-box 7 |
| SPACA1 | sperm acrosome associated 1 |
| SPAG8 | sperm associated antigen 8 |
| SPARCL1 | SPARC-like 1 (hevin) |
| SPG7 | spastic paraplegia 7 (pure and complicated autosomal recessive) |
| SPHKAP | SPHK1 interactor, AKAP domain containing |
| SPINK6 | serine peptidase inhibitor, Kazal type 6 |
| SPOCK2 | sparc/osteonectin, cwcv and kazal-like domains proteoglycan (testican) 2 |
| SPOCK3 | sparc/osteonectin, cwcv and kazal-like domains proteoglycan (testican) 3 |
| SPTA1 | spectrin, alpha, erythrocytic 1 (elliptocytosis 2) |
| SPTBN1 | spectrin, beta, non-erythrocytic 1 |
| SRGAP3 | SLIT-ROBO Rho GTPase activating protein 3 |
| SRGN | serglycin |
| SSX4 | synovial sarcoma, X breakpoint 4 |
| SSX4B | synovial sarcoma, X breakpoint 4B |
| ST13 | suppression of tumorigenicity 13 (colon carcinoma) (Hsp70 interacting protein) |
| ST5 | suppression of tumorigenicity 5 |
| ST6GAL1 | ST6 beta-galactosamide alpha-2,6-sialyltranferase 1 |
| ST6GALNAC1 | ST6 (alpha-N-acetyl-neuraminyl-2,3-beta-galactosyl-1,3)-N-acetylgalactosaminide alpha-2,6-sialyltransferase 1 |
| ST8SIA1 | ST8 alpha-N-acetyl-neuraminide alpha-2,8-sialyltransferase 1 |
| STAB2 | stabilin 2 |
| STAT5A | signal transducer and activator of transcription 5A |
| STIM2 | stromal interaction molecule 2 |
| STK17A | serine/threonine kinase 17a |
| STRADA | STE20-related kinase adaptor alpha |
| STRADB | STE20-related kinase adaptor beta |
| SUCLG1 | succinate-CoA ligase, alpha subunit |
| SULT1C3 | sulfotransferase family, cytosolic, 1C, member 3 |
| SUV39H2 | suppressor of variegation 3-9 homolog 2 (Drosophila) |
| SV2C | synaptic vesicle glycoprotein 2C |
| SVEP1 | sushi, von Willebrand factor type A, EGF and pentraxin domain containing 1 |
| SYCE1 | synaptonemal complex central element protein 1 |
| SYNE1 | spectrin repeat containing, nuclear envelope 1 |
| TAC1 | tachykinin, precursor 1 |
| TACR1 | tachykinin receptor 1 |
| TANK | TRAF family member-associated NFKB activator |
| TAS2R14 | taste receptor, type 2, member 14 |
| TAS2R4 | taste receptor, type 2, member 4 |
| TBC1D3F | TBC1 domain family, member 3F |
| TBC1D4 | TBC1 domain family, member 4 |
| TBRG1 | transforming growth factor beta regulator 1 |
| TCF12 | transcription factor 12 |
| TCF7L2 | transcription factor 7-like 2 (T-cell specific, HMG-box) |
| TCHH | trichohyalin |
| TCN2 | transcobalamin II |
| TEAD1 | TEA domain family member 1 (SV40 transcriptional enhancer factor) |
| TESC | tescalcin |
| TF | transferrin |
| TFAP2E | transcription factor AP-2 epsilon (activating enhancer binding protein 2 epsilon) |
| TFPI | tissue factor pathway inhibitor (lipoprotein-associated coagulation inhibitor) |
| TGDS | TDP-glucose 4,6-dehydratase |
| TGFA | transforming growth factor, alpha |
| THBD | thrombomodulin |
| THYN1 | thymocyte nuclear protein 1 |
| TIMP4 | TIMP metallopeptidase inhibitor 4 |
| TINAGL1 | tubulointerstitial nephritis antigen-like 1 |
| TJP2 | tight junction protein 2 (zona occludens 2) |
| TK2 | thymidine kinase 2, mitochondrial |
| TLR1 | toll-like receptor 1 |
| TLR6 | toll-like receptor 6 |
| TMC7 | transmembrane channel-like 7 |
| TMC8 | transmembrane channel-like 8 |
| TMED6 | transmembrane emp24 protein transport domain containing 6 |
| TMEM109 | transmembrane protein 109 |
| TMEM138 | transmembrane protein 138 |
| TMEM168 | transmembrane protein 168 |
| TMEM188 | transmembrane protein 188 |
| TMEM231 | transmembrane protein 231 |
| TMEM35 | transmembrane protein 35 |
| TMPRSS5 | transmembrane protease, serine 5 |
| TMSB4X | thymosin beta 4, X-linked |
| TNFAIP8 | tumor necrosis factor, alpha-induced protein 8 |
| TNFSF14 | tumor necrosis factor (ligand) superfamily, member 14 |
| TNNI3K | TNNI3 interacting kinase |
| TNRC6B | trinucleotide repeat containing 6B |
| TNXA | tenascin XA pseudogene |
| TNXB | tenascin XB |
| TOB1 | transducer of ERBB2, 1 |
| TOX4 | TOX high mobility group box family member 4 |
| TPK1 | thiamin pyrophosphokinase 1 |
| TRD@ | T cell receptor delta locus |
| TRDMT1 | tRNA aspartic acid methyltransferase 1 |
| TRHDE | thyrotropin-releasing hormone degrading enzyme |
| TRPM3 | transient receptor potential cation channel, subfamily M, member 3 |
| TSLP | thymic stromal lymphopoietin |
| TSPAN32 | tetraspanin 32 |
| TSPAN4 | tetraspanin 4 |
| TSPAN6 | tetraspanin 6 |
| TSPYL2 | TSPY-like 2 |
| TTC28 | tetratricopeptide repeat domain 28 |
| TTC33 | tetratricopeptide repeat domain 33 |
| TTLL11 | tubulin tyrosine ligase-like family, member 11 |
| TTLL3 | tubulin tyrosine ligase-like family, member 3 |
| TTYH1 | tweety homolog 1 (Drosophila) |
| TTYH2 | tweety homolog 2 (Drosophila) |
| TUBE1 | tubulin, epsilon 1 |
| TUSC3 | tumor suppressor candidate 3 |
| TUSC5 | tumor suppressor candidate 5 |
| TXN2 | thioredoxin 2 |
| TXNDC6 | thioredoxin domain containing 6 |
| TXNIP | thioredoxin interacting protein |
| TXNL4B | thioredoxin-like 4B |
| TYW3 | tRNA-yW synthesizing protein 3 homolog (S. cerevisiae) |
| U2AF1L4 | U2 small nuclear RNA auxiliary factor 1-like 4 |
| UBA52 | ubiquitin A-52 residue ribosomal protein fusion product 1 |
| UGT8 | UDP glycosyltransferase 8 |
| UPF2 | UPF2 regulator of nonsense transcripts homolog (yeast) |
| USP10 | ubiquitin specific peptidase 10 |
| USP25 | ubiquitin specific peptidase 25 |
| USP30 | ubiquitin specific peptidase 30 |
| USP34 | ubiquitin specific peptidase 34 |
| USP53 | ubiquitin specific peptidase 53 |
| UTP6 | UTP6, small subunit (SSU) processome component, homolog (yeast) |
| UTRN | utrophin |
| UTS2D | urotensin 2 domain containing |
| VAMP5 | vesicle-associated membrane protein 5 (myobrevin) |
| VGLL1 | vestigial like 1 (Drosophila) |
| VPRBP | Vpr (HIV-1) binding protein |
| VPS29 | vacuolar protein sorting 29 homolog (S. cerevisiae) |
| VPS4A | vacuolar protein sorting 4 homolog A (S. cerevisiae) |
| VSX1 | visual system homeobox 1 |
| WASH1 | WAS protein family homolog 1 |
| WASH3P | WAS protein family homolog 3 pseudogene |
| WBP4 | WW domain binding protein 4 (formin binding protein 21) |
| WBSCR22 | Williams Beuren syndrome chromosome region 22 |
| WHAMML1 | WAS protein homolog associated with actin, golgi membranes and microtubules-like 1 |
| WIF1 | WNT inhibitory factor 1 |
| WNT10B | wingless-type MMTV integration site family, member 10B |
| WSCD2 | WSC domain containing 2 |
| WWTR1 | WW domain containing transcription regulator 1 |
| XPNPEP2 | X-prolyl aminopeptidase (aminopeptidase P) 2, membrane-bound |
| YBX1 | Y box binding protein 1 |
| YEATS2 | YEATS domain containing 2 |
| ZC3H12C | zinc finger CCCH-type containing 12C |
| ZC3H13 | zinc finger CCCH-type containing 13 |
| ZC3H7A | zinc finger CCCH-type containing 7A |
| ZFAND5 | zinc finger, AN1-type domain 5 |
| ZFP36L1 | zinc finger protein 36, C3H type-like 1 |
| ZMYM5 | zinc finger, MYM-type 5 |
| ZNF132 | zinc finger protein 132 |
| ZNF160 | zinc finger protein 160 |
| ZNF224 | zinc finger protein 224 |
| ZNF28 | zinc finger protein 28 |
| ZNF283 | zinc finger protein 283 |
| ZNF383 | zinc finger protein 383 |
| ZNF432 | zinc finger protein 432 |
| ZNF485 | zinc finger protein 485 |
| ZNF503 | zinc finger protein 503 |
| ZNF529 | zinc finger protein 529 |
| ZNF532 | zinc finger protein 532 |
| ZNF561 | zinc finger protein 561 |
| ZNF562 | zinc finger protein 562 |
| ZNF57 | zinc finger protein 57 |
| ZNF585A | zinc finger protein 585A |
| ZNF596 | zinc finger protein 596 |
| ZNF614 | zinc finger protein 614 |
| ZNF676 | zinc finger protein 676 |
| ZNF708 | zinc finger protein 708 |
| ZNF709 | zinc finger protein 709 |
| ZNF711 | zinc finger protein 711 |
| ZNF790 | zinc finger protein 790 |
| ZNF83 | zinc finger protein 83 |
| ZP4 | zona pellucida glycoprotein 4 |
